# Work stressors associated with mental health, well-being and intention to leave among ambulance staff in England: an explanatory mixed-methods study

**DOI:** 10.1136/bmjopen-2026-119532

**Published:** 2026-07-09

**Authors:** Zoe Anchors, Rachel Arnold, Helen Nicholson, Sarah Voss, Nicola E Walsh, Sarah Black, Lee Moore

**Affiliations:** 1 Department of Health, University of Bath, Bath, UK; 2 Centre for Health and Clinical Research, University of the West of England, Bristol, UK; 3 SWASFT, Exeter, UK

**Keywords:** MENTAL HEALTH, Emergency Service, Hospital, Occupational Stress, Health Workforce

## Abstract

**Abstract:**

**Background and aim(s):**

Ambulance staff have the highest sickness absence, burnout and turnover intentions in the National Health Service, with work-related stress and anxiety identified as key drivers. Although multiple occupational stressors have been reported, it remains unclear which have the greatest impact. This study examined which work stressors are most strongly associated with mental health, well-being and intention to leave; whether these associations are explained by stress appraisals and mental rest; and which staff are most vulnerable to adverse outcomes.

**Methods:**

A mixed-methods, explanatory, sequential design, stress audit was conducted in one ambulance service in England. An online survey with ambulance staff (n=420) assessed key stress elements (work stressors, stress appraisals, mental rest) and key outcomes (depression, well-being, intention to leave). Semistructured interviews with staff (n=8) explored the findings of the survey in more depth. Linear regression analyses examined relationships between predictors (eg, stressors) and outcomes (eg, well-being) and analysis of variances and t-tests explored differences between groups (eg, gender). A thematic analysis of qualitative data was conducted.

**Results:**

Quantitative and qualitative findings suggest that stress related to manager support (ie, upper management prioritising efficiency over staff needs), workplace demands (ie, organisational demands creating extra burden), poor relationships (eg, with non-peer staff) and constant change had the most negative impact on staff. Low mental rest and threat appraisals (ie, feeling ‘overwhelmed’ by work demands) were strong predictors of all negative outcomes, alongside cumulative stress exposure and negative emotional responses. Longer tenure, supervisory roles, mixed urban–rural working and male gender were associated with greater risk of poorer outcomes.

**Conclusions:**

This study offers a comprehensive and novel insight into the stress experiences of ambulance staff. Findings underscore the need for theoretically-informed, evidence-based interventions that integrate organisational reform and individual support strategies to mitigate sickness absence, safeguard well-being and prevent attrition.

**STRENGTHS AND LIMITATIONS OF THIS STUDY**

- The mixed-methods stress audit provides a holistic, actionable approach to understanding work stress and informing targeted staff well-being interventions.
- The study was conducted in one ambulance service in England, limiting generalisability to other UK or international services.
- While mixed-methods in nature, the cross-sectional design captured only a snapshot of work stress at one point in time.
- Due to the complexity of stressors, interactions between organisational stressors and individual factors may be more nuanced than presented.

## Introduction

 Ambulance staff report the highest burnout (~40%) in the UK’s National Health Service (NHS),^1^ with only a third feeling they have good work–life balance.^2^ They also have the highest sickness rates, largely driven by work stress.^3^ Staff leaver and turnover rates in ambulance services in England have increased since 2019,^4^ and burnout and poor well-being reduce the safety and quality of care,^5^ including prehospital care.^6^ High turnover, combined with rising demand, contributes to delayed response times and avoidable patient harm, including increased mortality^7^ and poorer patient experience.^8^ Most ambulance staff (91%) have experienced mental ill health,^9^ and symptoms linked to work stress can be long-lasting.^10^ Although NHS organisations report efforts to improve staff work–life balance, ambulance staff are the least likely to agree this support exists.^11^ Evidence for effective interventions to reduce sickness absence^12^ and improve mental health and well-being in this workforce remains limited.^13^

To better support ambulance staff with occupational stress and implement more tailored and effective interventions,^14 15^ this study aimed to conduct a stress audit. Stress audits provide valuable information that organisations can use to proactively address work-stress and create a healthier work environment.^16^ Specifically, a stress audit can help: (1) identify which stressors (ie, demands, pressures) have the most negative impact on outcomes (eg, health), (2) understand why these effects occur (eg, via appraisals, poor mental rest) and (3) recognise which employees are most ‘at risk’. Previous research has identified several stressors encountered by ambulance staff including: organisational demands (eg, changing shifts); excessive workload; low job autonomy; management pressure; poor supervisor support; and traumatic incidents, bullying and unacceptable violence.^6 9 11 17^ However, it is acknowledged that the contributing factors to ambulance staffs’ strain are complex^18^; and it is not well documented which have the greatest impact (eg, on intention to leave, mental ill-health).

The second aim of a stress audit is to understand how and why stressors have an impact. This study examined two mechanisms: stress appraisal and mental rest. In transactional stress theory,^19^ appraisal refers to individuals’ evaluations of important situations.^20^ When coping resources are limited, situations are more likely to be appraised as threats (demands exceed resources) rather than challenges (resources meet or exceed demands). Threat appraisals are linked to poorer performance and mental health,^21 22^ yet have received limited attention in ambulance research, highlighting the need to measure them here. Mental rest is another potential mechanism. In other high-pressure fields (eg, elite sport), it is a key recovery factor and predicts outcomes such as depression and well-being.^23^ Ambulance staff have described the need to mentally detach from work to cope with stress,^24^ but mechanisms explaining how work stress affects this group, particularly mental rest, remain underexplored.

The third aim of the stress audit is to identify which individuals or groups are most ‘at risk’ of negative stress-related consequences. Some attention has been given to the well-being of students, new recruits, experienced staff and male employees in an ambulance context,^25^,^27^ and when examining burnout specifically, those individuals most at risk were full-time male employees with more than 10 years’ experience and employed in a paramedic position.^27^ Evidence is mixed about the impact of work location on the well-being of ambulance staff.^28 29^ Further research is therefore required to better understand which ambulance staff are most vulnerable (eg, Band level, role, work location, gender) to identify who is most at risk and in need of intervention.

This stress audit was informed by two frameworks: (1) transactional stress theory,^19^ which focuses on how individuals appraise and cope with situations and associated emotions and (2) the transdisciplinary model of stress.^30^ This latter model conceptualises stress as arising not only from individual appraisal processes, but also from interactions between organisational structures, social dynamics, and environmental demands, making it particularly relevant to high-pressure healthcare settings such as ambulance services. Guided by these models, the study aimed to: (1) identify which stressors were most strongly associated with ambulance staff’s mental health, well-being, and intention to leave; (2) understand why these effects occurred (ie, via stress appraisals and mental rest) and (3) determine which groups were most at risk of negative stress-related outcomes.

## Methods

This study used a sequential explanatory mixed-methods design, beginning with an online survey followed by interviews. The qualitative phase was used to explain and expand on the quantitative findings,^31^ with survey results guiding purposive sampling and targeted recruitment of at-risk groups.^32^

### Quantitative component

#### Participants

Ambulance staff that included frontline, clinical hub and dispatch and support workers were recruited from one NHS ambulance service in England. The survey was completed by 420 staff representing 7% of the eligible workforce within the service. Their characteristics are shown in table 1. Given the high workload of this population, sample size was driven by staff availability and study resources rather than statistical power alone.^33^ Although a larger sample would improve precision, the aim was to identify potential stress-management interventions to inform future national work. Importantly, the sample exceeded the recommended minimum of 250 participants for stable correlations.^34^

**Table 1:** Survey participant and work characteristics (n=420)

Characteristic	Statistic (n, %)
Gender	
Female	160 (38.1)
Male	188 (44.8)
Missing	72 (17.1)
Age	
18–24 years	21 (5.0)
25–34 years	134 (31.9)
35–44 years	81 (19.3)
45–55 years	90 (21.4)
55 years and over	28 (6.7)
Missing	66 (15.7)
Primary geographical work location	
Rural area	78 (18.6)
Urban area	119 (28.3)
Mixed area	160 (38.1)
Missing	63 (15.0)
Length of time in role	
<2 years	72 (17.1)
2–5 years	78 (18.6)
5–10 years	110 (21.2)
>10 years	89 (21.2)
Missing	71 (16.9)
Band level	
≤3	84 (20.0)
4	35 (8.3)
5	71 (16.9)
6	119 (28.3)
7	32 (7.6)
≥8	12 (2.9)
Missing	67 (16.0)
Role	
Frontline ambulance	241 (57.4)
Clinical hubs and dispatch	57 (13.6)
Support role	40 (9.5)
Other	19 (4.5)
Missing	63 (15.0)
Working hours	
Full time	286 (68.1)
Part time	57 (13.6)
Bank	14 (3.3)
Missing	63 (15.0)

‘Missing’ refers to participants who completed the survey but did not provide a response for that specific item.

Bank refers staff who work on a flexible, shift-by-shift basis (‘bank’ work) rather than being permanently employed on a fixed contract or roster.

#### Procedure

Following research governance approval, ambulance staff were recruited. The survey was pilot-tested with two authors, one advisory panel member, and three staff (a paramedic, clinical hub worker, and support worker). It was revised based on feedback (eg, including Bank staff as a distinct staff group) and formatted in Qualtrics. A second pilot with a Research Fellow checked logic, routing, and timing, leading to minor wording and question-order changes.

The final survey was distributed electronically via a short Vimeo clip in routine news bulletins and on staff social media (eg, a closed Facebook group). The opening page included a link to the participant information sheet outlining the General Data Protection Regulation, confidentiality, and withdrawal rights, and participants were informed that completing the survey indicated consent. At the end, respondents could leave an email if willing to be interviewed. The survey was open from 16 May 2023 to 21 June 2023.

#### Measures

##### Work stressors

Work stressors were measured using the refined 25-item Health and Safety Executive Management Standards Indicator Tool,^35^ which assesses six work design areas linked to poorer health, lower productivity, and higher sickness absence.^36^ The areas are: (1) Demands (eg, ‘I have unachievable deadlines’); (2) Control (eg, ‘I have some say over the way I work’); (3) Support (eg, ‘I am given supportive feedback’); (4) Relationships (eg, ‘I am subject to bullying at work’); (5) Role (eg, ‘I am clear about my responsibilities’) and (6) Change (eg, ‘Staff are consulted about change’). Items were rated on a 5-point scale (1=strongly disagree to 5=strongly agree), with higher scores indicating better management standards and lower stress. Negatively worded items were reverse scored. The tool has established validity,^37^ and subscales showed acceptable reliability (demands=0.70, control=0.84, manager support=0.88, peer support=0.83, relationships=N/A due to two items, role=0.78, change=0.82).

##### Stress appraisals

Two self-report items from the Cognitive Appraisal Ratio were adapted to assess evaluations of work demands and coping resources.^38^ Demand appraisal was measured with: ‘How demanding do you generally find it at work?’, and resource appraisal with: ‘How able are you to cope with the demands you encounter at work?’ Both used a 6-point scale (1=not at all to 6=extremely). A stress appraisal score was calculated by subtracting demands from resources (range −5 to 5), where zero or positive values indicated a challenge appraisal (resources match/exceed demands) and negative values indicated a threat appraisal (demands exceed resources). This measure is widely used in high-pressure contexts.^21^

##### Mental rest

Mental rest was assessed using an adapted measure of current psychological rest levels.^39^ After reading brief descriptions of poorly mentally rested staff (eg, ‘feeling tired’, ‘not valuing my job’, ‘lacking motivation’) and well-rested staff (eg, ‘feeling fresh’, ‘valuing my job’, ‘highly motivated’), participants rated their mental rest over the past month on a 5-point scale (1=poorly mentally rested to 5=well mentally rested). Higher scores indicated better mental rest. This measure has been used in other high-pressure settings (eg, elite sport^23 39^).

##### Depression

The Patient Health Questionnaire-2 (PHQ-2^40^) assessed depression using two items: ‘Having little interest or pleasure in doing things’ and ‘Feeling down, depressed, or hopeless.’ Responses were rated on a 4-point scale (0=not at all to 3=nearly every day). Item scores were summed (range 0–6), with higher scores indicating greater depressive symptomology. The PHQ-2 has been validated in prior research.^41^

##### Well-being

The WHO Well-Being Index (WHO-5^42^) measured well-being using five items (eg, ‘I have felt cheerful and in good spirits’). Items were rated on a 6-point scale (0=at no time to 5=all the time), with higher scores indicating better well-being. WHO-5 scores were calculated by summing item responses (0–5 each) to produce a total score (range 0–25), which was then multiplied by 4 to generate a percentage score (range 0–100), with higher scores indicating better well-being. The measure has established validity^43^ and showed excellent reliability in this study (α=0.91).

##### Intention to leave

Participants rated their agreement with one item (‘I want to leave the ambulance service’) on a 5-point scale (1=strongly disagree to 5=strongly agree), with higher scores indicating greater intention to leave. Single-item measures of turnover intention are commonly used in occupational health research, particularly in applied settings where survey brevity is required. For example, they have been used in studies of occupational stress among midwives^44^ and evidence suggests they show acceptable validity and strong associations with multi-item measures of job attitudes and withdrawal cognitions.^45^ It is acknowledged that single-item measures do not permit assessment of internal consistency reliability and may be more susceptible to measurement error.

##### Work and demographic variables

The survey captured primary work location (urban, rural or mixed), time in current role (years), Band (3/4=emergency care assistant; 5=newly qualified paramedic; 6=paramedic >2 years; 7/8=leadership/management), working capacity (full-time, part-time or bank), role type (frontline, clinical hub/dispatch or support) and demographics (age, gender).

### Data analysis

Quantitative data were analysed in SPSS V.29. Missing data were present across several variables, and analyses were conducted using all available data for each variable, resulting in varying denominators. Means, SDs and intercorrelations were computed for all stress related and outcome variables. Bivariate linear regressions examined relationships between stress-related variables (stressors, appraisals, mental rest) and outcomes (depression, well-being, intention to leave). Standardised beta coefficients were used as indicators of effect size in regression models and were interpreted in line with established benchmarks for small, medium, and large effects.^46^ Standardised coefficients are comparable to correlation-based effect sizes and can therefore be interpreted in a similar manner.^47^ Alpha was set at p<0.05. These analyses aimed to (1) identify which stressors were most strongly linked to mental health, well-being, and intention to leave and (2) examine why these effects occurred (ie, whether stress appraisals and/or mental rest predicted outcomes). Independent t-tests and one-way analysis of variances (ANOVAs) were then used to assess differences in stress-related variables and outcomes across work and demographic factors (age, gender, time in role, Band, role type, work capacity), helping to identify staff at greater risk of negative stress-related outcomes. Cohen’s d was used to quantify effect sizes for independent samples t-tests, while omega-squared (ω²) effect size estimates were reported for all ANOVA analyses.

### Qualitative component

#### Participants

Eight participants took part in the qualitative component (seven ambulance staff, including one manager and one occupational health professional). Their characteristics are shown in table 2.

**Table 2:** Interview participants and their demographic and work characteristics (n=8)

Characteristic	Participant number							
	1	2	3	4	5	6	7	8
Gender	Female	Female	Male	Male	Male	Male	Female	Male
Age (years)	NR	25–34	45–55	45–55	45–55	45–55	35–44	55+
Primary work location	Urban	Mixed	Urban	Rural	Rural	Mixed	Mixed	Mixed
Length of time in role (years)	N/A	5–10	5–10	5–10	10+	10+	<2	10+
Band level	N/A	6	6	6	6	6	8	6
Role	OHP	Paramedic	Paramedic	Paramedic	Paramedic	Paramedic	Manager	Paramedic
Work capacity	Full time	Full time	Full time	Full time	Full time	Bank	Full time	Bank

N/A, not available; NR, not reported; OHP, occupational health professional.

#### Procedure

After the quantitative phase, we identified the most impactful stressors in the sample and invited individuals scoring highly on these (eg, work demands) to interview. Key survey findings (ie, influential stress-related variables and vulnerable staff groups) informed the interview guide. Of the 177 staff who agreed to be contacted, 22 were emailed with study details and an invitation to participate. Eight agreed, provided informed consent, and received a £15 gift card. Interviews were conducted online, recorded, and transcribed verbatim. They lasted 53–72 min (M=62.13, SD=6.29). All interviews were conducted by the lead author who has extensive experience in interviewing NHS professionals reporting occupational stress. The interviewer’s background and role were made explicit to participants prior to the start of the interview. A reflexive approach was adopted, recognising that the interviewer’s non-clinical position and prior experience may have shaped data collection and interpretation; these influences were considered throughout, including during analysis through ongoing reflection and team discussion.

#### Interview guide

Semistructured interviews explored key survey findings. After rapport building, participants were shown the most negatively impactful stressors and asked how and why these might affect outcomes. They also discussed how they appraised each stressor (eg, challenge or threat), their mental rest, and the impact of both. Participants were then informed about the most vulnerable groups and asked why these staff might be at greater risk. The interview guide (see online supplemental materials) was adapted for the manager and occupational health professional to examine identification of work stress and support provision.

#### Data analysis

Qualitative data were analysed in NVivo V.14 using thematic analysis^48^ with a hybrid abductive approach combining inductive, data-driven coding^49^ and a deductive template of a priori codes.^50^ This allowed survey findings to guide deductive coding while also enabling themes to be generated directly from the data. The occupational health professional interview was included to provide an organisational and occupational health perspective on workforce well-being and retention. This interview was analysed within the same thematic framework as ambulance staff data and used to contextualise and triangulate theme development rather than to generate separate thematic structures.

The lead author (a non-clinical Research Fellow) read all transcripts, made initial notes and generated descriptive and latent codes linked to the study aim. To enhance reflexivity and minimise individual bias, a second author (a clinical academic paramedic) coded 25% of transcripts (n=2), and coding was compared and agreed. Themes capturing key patterns related to the study aim were developed by clustering codes and identifying shared meaning. Three authors reviewed and refined the themes, merging overlapping ones and finalising definitions, names and illustrative quotes. Participant numbers are used in reporting to protect confidentiality.

This sample size was guided by principles of analytic depth and information power rather than statistical representativeness.^51^ Consistent with reflexive thematic analysis, data saturation was not treated as a formal criterion^52^; however, analysis indicated a high degree of informational redundancy, with no substantially new themes being generated during later interviews. Evidence from qualitative health research further supports that thematic saturation or redundancy can be reached within relatively small samples in homogeneous populations.^53^ Our sample was relatively homogeneous in that participants worked within the same organisation and, based on findings from the quantitative phase, reported similarly high scores on key workplace stressors such as work demands. Rigour and quality were supported by following recommended procedures for hybrid thematic analysis^54^ and mixed-methods research.^55^ For example, in the qualitative phase, cross-checking and reviewing codes among researchers strengthened inter-rater reliability and the credibility of themes. Comparable rigour was applied in the quantitative phase through pilot testing. The use of transactional stress theory^19^ and the transdisciplinary model of stress^30^ provided a strong theoretical foundation for integrating multiple data types to address the complexity of occupational stress.

### Patient and public involvement

This research was conducted with NHS ambulance staff and did not directly involve patients or members of the public as study participants. However, patient and public involvement was incorporated through an advisory panel comprising emergency care clinicians, academic researchers and patient contributors. The panel met on two occasions and contributed to the development of the interview topic guide, interpretation of findings and dissemination plans.

## Results

### Quantitative results

#### Descriptive statistics

Online supplemental table 1 displays the means, SDs and intercorrelations. Pearson and bivariate correlation analyses revealed that all stress-related variables were significantly associated with outcomes.

#### Regression results

Table 3 displays the results of the bivariate linear regression analyses. All stress-related variables significantly predicted symptoms of depression. The largest effect sizes were for workplace demands (β=−0.43) and mental rest (β=−0.42), indicating that participants who experienced greater workplace stress linked to job demands, and participants who were less mentally rested, reported greater depression symptomology.

**Table 3:** Linear regression models examining if stress-related variables significantly predicted outcomes

	R^2^	B	SE	β	t	P value	95% CI
Depression							
HSE demands	0.19	−0.90	0.10	−0.43	−9.29	<0.001	−1.09 to −0.71
HSE control	0.05	−0.43	0.10	−0.23	−4.55	<0.001	−0.62 to −0.25
HSE manager support	0.10	−0.56	0.09	−0.32	−6.50	<0.001	−0.73 to −0.39
HSE peer support	0.07	−0.61	0.12	−0.26	−5.24	<0.001	−0.84 to −0.38
HSE relationships	0.13	−0.65	0.09	−0.36	−7.37	<0.001	−0.82 to −0.47
HSE role	0.06	−0.48	0.10	−0.24	−4.77	<0.001	−0.68 to −0.28
HSE change	0.06	−0.47	0.09	−0.25	−5.00	<0.001	−0.65 to −0.28
Stress appraisal	0.16	−0.41	0.05	−0.40	−8.33	<0.001	−0.50 to −0.31
Mental rest	0.17	−0.58	0.07	−0.42	−8.82	<0.001	−0.71 to −0.45
Well-being							
HSE demands	0.14	9.82	1.28	0.37	7.68	<0.001	7.31 to 12.33
HSE control	0.14	9.10	1.16	0.38	7.83	<0.001	6.81 to 11.39
HSE manager support	0.17	9.25	1.07	0.41	8.69	<0.001	7.16 to 11.35
HSE peer support	0.12	10.26	1.45	0.35	7.08	<0.001	7.40 to 13.10
HSE relationships	0.03	3.64	1.19	0.16	3.06	<0.01	1.30 to 5.98
HSE role	0.09	7.58	1.27	0.30	5.98	<0.001	5.09 to 10.07
HSE change	0.17	9.94	1.13	0.42	8.77	<0.001	7.71 to 12.17
Stress appraisal	0.22	6.14	0.60	0.47	10.21	<0.001	4.96 to 7.32
Mental rest	0.40	11.32	0.73	0.63	15.56	<0.001	9.89 to 12.75
Intention to leave							
HSE demands	0.24	−0.78	0.07	−0.49	−10.76	<0.001	−0.92 to −0.63
HSE control	0.13	−0.52	0.07	−0.35	−7.29	<0.001	−0.65 to −0.38
HSE manager support	0.23	−0.64	0.06	−0.48	−10.45	<0.001	−0.77 to −0.52
HSE peer support	0.14	−0.67	0.09	−0.37	−7.79	<0.001	−0.84 to −0.50
HSE relationships	0.23	−0.66	0.06	−0.47	−10.39	<0.001	−0.79 to −0.54
HSE role	0.15	−0.60	0.07	−0.39	−8.10	<0.001	−0.74 to −0.45
HSE change	0.18	−0.61	0.07	−0.42	−8.90	<0.001	−0.74 to −0.47
Stress appraisal	0.08	−0.23	0.04	−0.29	−5.84	<0.001	−0.31 to −0.15
Mental rest	0.19	−0.47	0.05	−0.43	−9.19	<0.001	−0.57 to −0.37

HSE, Health and Safety Executive.

All stress-related variables significantly predicted well-being. The largest effect sizes were for mental rest (β=0.63); stress appraisal (β=0.47); manager support (β=0.41); and change (β=0.42). Thus, participants who reported being less mentally rested, appraising their job as more of a threat, and experiencing greater workplace stress linked to manager support and change were more likely to report poorer well-being.

All stress-related variables significantly predicted intention to leave. The largest effect sizes were for job demands (β=−0.49); manager support (β=−0.48); relationships (β=−0.47); mental rest (β=0.43) and change (β=−0.42). Thus, participants who reported experiencing greater workplace stress linked to job demands, manager support, relationships and change, as well as being less mentally rested, reported a higher intention to leave.

#### Difference test results

Table 4 presents the one-way ANOVA results examining differences in outcomes and stress-related variables across work and demographic characteristics. Significant post hoc LSD t-tests (p<0.05) are summarised below. Table 5 shows the independent t-test results comparing males and females. Means and SD for all variables are provided in online supplemental tables 2 and 3.

**Table 4:** Results of one-way ANOVAs examining differences in outcomes and stress-related variables for work and demographic characteristics

	Characteristics	F	df	P value	ω²
Outcomes					
Depression	Age	1.34	4	0.254	0.004
	Location	0.06	2	0.939	−0.005
	Experience	5.04	3	**0.002**	0.034
	Band level	1.51	5	0.185	0.004
	Role type	1.30	3	0.276	0.002
	Work capacity	0.80	2	0.448	−0.001
Well-being	Age	1.35	4	0.252	0.004
	Location	4.31	2	**0.014**	0.018
	Experience	1.52	3	0.208	0.004
	Band level	1.65	5	0.147	0.009
	Role type	3.12	3	**0.026**	0.018
	Work capacity	0.47	2	0.627	−0.001
Intention to leave	Age	0.30	4	0.878	−0.008
	Location	2.11	2	0.122	0.006
	Experience	11.92	3	**<0.001**	0.086
	Band level	7.46	5	**<0.001**	0.084
	Role type	1.75	3	0.156	0.006
	Work capacity	2.37	2	0.095	0.008
Stress-related variables					
HSE demands	Age	0.27	4	0.900	−0.008
	Location	4.65	2	**0**.**010**	0.020
	Experience	6.56	3	**0**.**000**	0.046
	Band level	2.01	5	0.077	0.014
	Role type	6.79	3	**0**.**000**	0.046
	Work capacity	0.29	2	0.750	−0.004
HSE control	Age	1.19	4	0.316	0.002
	Location	6.63	2	**0.001**	0.021
	Experience	10.11	5	**0.000**	0.024
	Band level	10.11	5	**0.000**	0.114
	Role type	16.21	3	**0.000**	0.113
	Work capacity	3.99	2	0.019	0.016
HSE manager support	Age	1.08	4	0.369	0.001
	Location	7.54	2	**0.001**	0.035
	Experience	7.07	3	**0.000**	0.050
	Band level	5.59	5	**0.000**	0.061
	Role type	8.71	3	**0.000**	0.061
	Work capacity	7.36	2	**0.001**	0.034
HSE peer support	Age	1.27	4	0.281	0.003
	Location	0.23	2	0.790	−0.004
	Experience	8.48	3	**0**.**000**	0.060
	Band level	1.86	5	0.100	0.012
	Role type	2.87	3	**0.037**	0.017
	Work capacity	1.09	2	0.338	0.000
HSE relationships	Age	2.10	4	0.080	0.012
	Location	2.01	2	0.135	0.006
	Experience	8.13	3	**0.000**	0.058
	Band level	2.06	5	0.070	0.015
	Role type	11.05	3	**0.000**	0.078
	Work capacity	1.73	2	0.178	0.004
HSE role	Age	1.73	2	0.178	−0.006
	Location	0.44	4	0.776	0.000
	Experience	1.08	2	0.342	0.002
	Band level	1.27	3	0.285	−0.010
	Role type	0.32	5	0.900	−0.003
	Work capacity	0.66	3	0.579	0.005
HSE change	Age	2.81	4	**0.026**	0.020
	Location	21.54	2	**0.000**	0.103
	Experience	6.11	3	**0.000**	0.042
	Band level	10.84	5	**0.000**	0.112
	Role type	20.53	3	**0.000**	0.141
	Work capacity	4.78	2	**0.009**	0.021
Stress appraisal	Age	1.65	2	0.161	0.007
	Location	1.49	4	0.226	0.003
	Experience	1.34	2	0.261	0.003
	Band level	1.51	3	0.185	0.007
	Role type	0.20	5	0.900	−0.007
	Work capacity	2.24	3	0.108	0.007
Mental rest	Age	3.24	4	**0.012**	0.025
	Location	8.05	2	**0.000**	0.038
	Experience	2.18	3	0.091	0.010
	Band level	5.66	5	**0.000**	0.062
	Role type	7.52	3	**0.000**	0.052
	Work capacity	1.27	2	0.283	0.001

Values in bold are significant at p<0.05.

ANOVA, analysis of variances; HSE, Health and Safety Executive.

**Table 5:** Results of independent t-tests to examine differences in outcomes and stress-related variables for gender

	t	df	P value	d
Outcomes				
Depression	−2.06	346	**0.040**	−0.22
Well-being	0.30	346	0.765	0.03
Intention to leave	−3.26	346	**0**.**001**	−0.35
Stress-related variables				
HSE demands	2.51	346	0.012	0.27
HSE control	0.56	346	0.575	0.06
HSE manager support	3.09	346	0.002	0.33
HSE peer support	1.84	346	0.067	0.20
HSE relationships	2.24	346	0.026	0.24
HSE role	3.31	346	0.001	0.36
HSE change	2.64	346	0.009	0.28
Stress appraisal	−0.95	346	0.344	−0.10
Mental rest	0.49	346	0.624	0.05

Values in bold are significant at p<0.05.

HSE, Health and Safety Executive.

Regarding outcomes (eg, well-being), participants who had worked longer in their role (ie, >2 years) reported higher depression symptomology than those who reported less time in their role (ie, <2 years). Participants who had spent less time in their role (ie, <2 years) also reported lower intention to leave than whose who had worked longer in their role (ie, >2 years). Band 6s reported higher intention to leave than Band 7s and Bands ≤5. Staff working in mixed urban–rural areas reported lower well-being than those in urban locations only, and front-line staff reported lower well-being than those in clinical hubs/dispatch or support roles. Male staff reported more depression symptoms and higher intention to leave than female staff.

In terms of stress-related variables, participants who had been in their current role longer reported more work-stress linked with demands, control, manager support, peer support, relationships and change than participants who had been in their current role for less time. For example, staff working for 5–10 years in their current role reported more workplace stress related to demands than those working for less than 2 years. Moreover, relative to those working in rural or urban locations only, participants working in mixed locations reported more work-stress related to demands, control, manager support and change. Additionally, younger participants (ie, 25–34 years) reported more work-stress linked to change than older participants (ie, 45–54 years).

Furthermore, Band 6s reported more work-stress related to manager support and change than all other Bands, and Band 6s also reported more work-stress linked with control than Bands 7 and 8. Front-line staff reported greater work stress related to control, manager support, and change than those in clinical hubs/dispatch or support roles, but lower relationship-related stress. Bank workers (qualified staff who work on a flexible, ad hoc shift basis rather than a permanent contracted rota (a preplanned schedule of shifts or roster)) reported more stress linked to control and manager support than full-time or part-time staff. Male staff reported more stress related to demands, manager support, relationships, role and change than female staff.

No significant differences were identified for stress appraisal. However, older staff (ie, 35–54 years) reported poorer mental rest than younger staff (ie, 25–34 years); staff in mixed locations reported poorer mental rest than staff in urban areas; Band 6s reported lower mental rest than all other Bands; and front-line staff reported poorer mental rest than staff from clinical hub/dispatch or support roles. No gender differences were found for mental rest.

#### Summary

Job demands, manager support, relationships and change were the stressors most strongly linked to outcomes. Stress appraisals were most strongly associated with well-being, whereas mental rest showed consistently strong associations across all outcomes, including depression, well-being and intention to leave. Staff at greater risk of negative stress-related outcomes (eg, poorer mental rest, well-being and depression and higher intention to leave) included those longer in role, Band 6 staff, males, front-line workers, those in mixed rural/urban locations and bank staff. These findings informed sampling criteria and the interview guide for the qualitative phase.

### Qualitative findings

**Figure 1:**
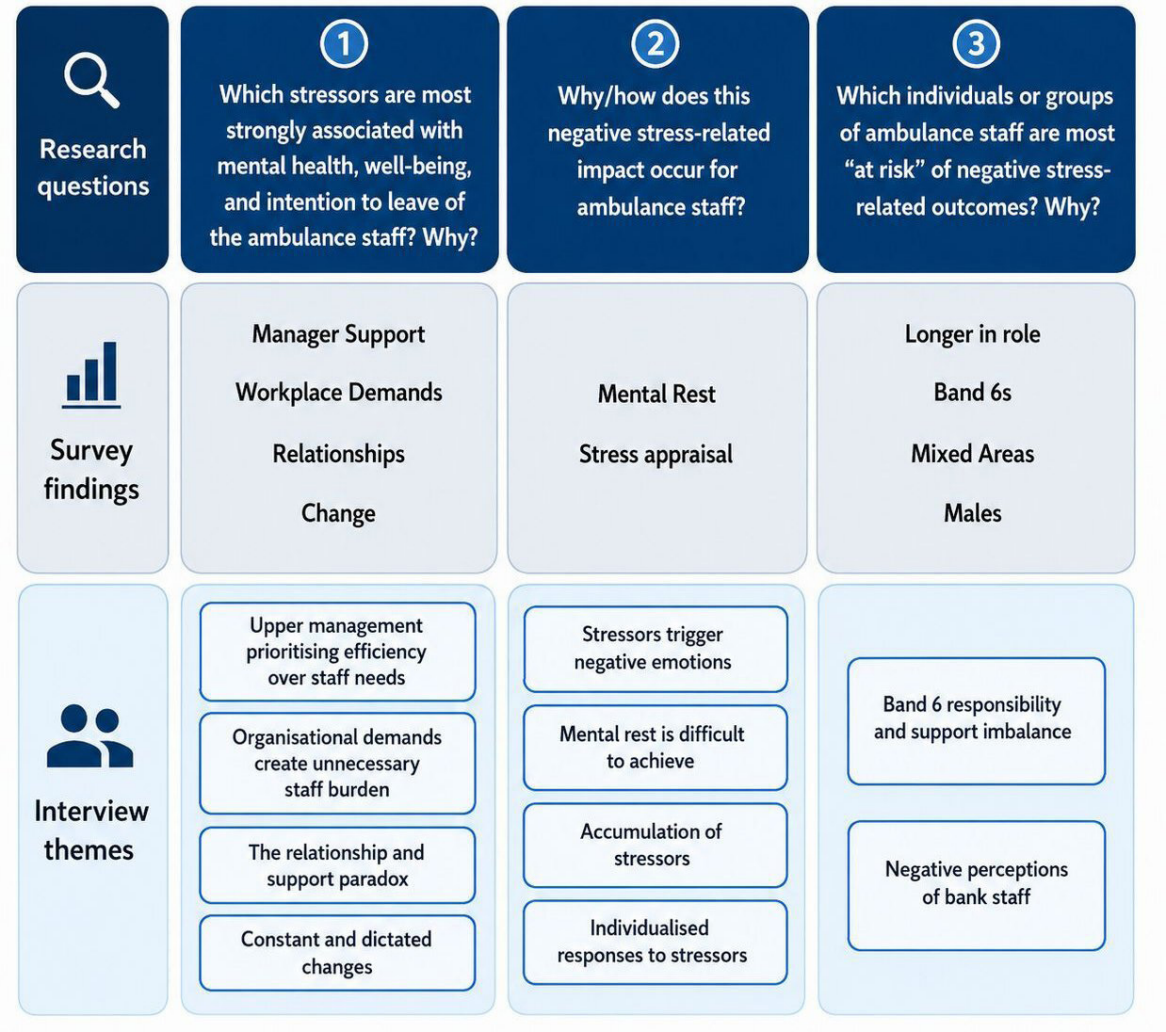
Explanatory thematic analysis of the key survey findings.

The thematic analysis generated 10 themes explaining the key survey findings, presented in relation to each research question (see figure 1).

### Why are the identified stressors demanding for ambulance staff?

#### Upper management prioritising efficiency over staff needs

The first theme reflected staff perceptions that upper management prioritised efficiency over well-being, pressuring them to work harder. Organisational changes were seen as aimed at productivity rather than improving conditions:

We keep having new policies brought in, changes to our working conditions, and we’re often told, oh, this is temporary because of the demand, and they’re then looking at the next way to get another job out of the crew, or to speed things up…. and none of these policies that are coming into place, ever feel positive for the staff. [Participant 2]

Participants also felt the stressors encountered were demanding because management support felt superficial, not well resourced, and staff perceived inadequate support. Indeed, some staff did not know who their managers were or had rarely met with them.

#### Organisational demands create unnecessary staff burden

Organisational demands such as the delivery cell (ie, a local ambulance service unit that coordinates and delivers frontline emergency response within a defined area) and poor equipment (eg, ill-functioning ambulances) negatively impacted staff stress and morale. The delivery cell, created to improve resource availability, required constant communication about job timings. Staff felt “hassled and harassed” [Participant 2] by what they described as a “draconian” system [Participant 8]. Although intended to increase productivity, the pressure to check in regularly was reported to undermine concentration:

You had to keep calling them [Delivery Cell] back every 15 minutes, to justify why you needed another 15 minutes. You couldn’t just say: ‘Right, I’ve got a long patient record to complete. I need to make two phone calls and a referral. I just need half-an-hour or so.’ And every 15 minutes, you had to keep picking the radio up, breaking your train of concentration. [Participant 8]

In addition to the delivery cell, lack of equipment or poorly functioning equipment severely impacted staff stress. Examples shared of such issues included ambulances breaking down, response vehicles not being available and poorly stocked ambulances.

#### The relationship and support paradox

All participants reported positive interactions with their working peers with no evidence of conflict or bullying. These supportive relationships, particularly for paramedics with fellow crew members, were described as a coping mechanism for the demands at work, and increased their likelihood of remaining in their roles:

I've got a good supportive team around me that we work with, the other paramedics and emergency care assistance, so for me to leave it would be a big thing. [Participant 4]

While some relationships were supportive and helped buffer stress, participants felt that expected support in other areas was absent or ineffective, increasing stress. Several described poor relationships with Emergency Department staff, recalling instances where paramedics felt their clinical judgement and decisions prior to hospital admission were not respected.

#### Constant and dictated changes

A final theme concerned the sense of constant, ongoing change. Staff expressed a desire for stability, describing frequent changes as demanding because they were rapid, poorly communicated, lacked evidence, and were ‘dictated’ to enhance productivity:

They’ve just done a load now, where they review working practices—as in start and finish times, shift length, number of nights, rota patterns…They just want to get the biggest bang for their buck. They’re trying to cut costs, improve service. There’s quite a lot of dictation. The hospitals need to get a grip of it. The end result is people leave… It caused me to go bank. [Participant 8]

### How do the demands negatively impact ambulance staff?

#### Stressors trigger negative emotions

Stressors triggered negative emotions that affected well-being and performance. The most emotive stressors were service changes, end-of-shift demands and primary care call-outs. Poorly communicated changes produced frustration and anger, while delayed shift endings caused apprehension. These emotions were seen as increasing stress, intention to leave, reduced performance, and apathy. Primary care call-outs also evoked anger, guilt, and embarrassment, as staff felt their role was to attend life-threatening emergencies rather than low-acuity cases. When these emotions had to be suppressed during a primary call out, stress intensified for this paramedic:

Anger, frustration, doubting the whole purpose of your job within the confines of the ambulance cab because the moment you step outside the ambulance cab you are a professional and you leave all of those biases and feelings locked in the cab. The act of doing that and locking all that [negative emotions] away, that in itself can add to the stress as well. [Participant 3]

#### Mental rest is difficult to achieve

Achieving cognitive and emotional detachment was described as extremely difficult due to the nature of ambulance work. Participants said that times when they could have rested were instead spent dwelling on traumatic incidents and questioning clinical decisions. Mental rest was hard to achieve both during shifts and outside work, with certain locations triggering distress years later and rumination continuing on the way to work:

Where I just brushed stuff off before, now I do tend to ruminate…Stuff will pop into my head, when it doesn’t need to, about work…Often not at work, because I’ll be dealing with something else, which pushes it all out of the way. So, personally, mental rest for me at the moment is not brilliant. [Participant 8]

One participant reported declining ability to mentally rest over time, improving only after moving to part-time work. Lack of mental rest was linked to low energy, fatigue, poor sleep and feelings of instability, with one participant noting it also led them to place greater responsibility on patients rather than leading care decisions.

#### Accumulation of stressors

The penultimate theme to explain how the demands were perceived to negatively impact ambulance staff was linked to the accumulation of stressors. As an example, one participant described the accumulation of service changes:

It was almost like each individual change probably wasn’t that big a deal in isolation, but it was that accumulative effect of all those changes that were the issue…. That each individual thing was never a big stressor, but actually that accumulative effect, so much change in such short space of time and for very, very much the wrong reasons was the biggest hit to me, the biggest stressor. [Participant 6]

The effect of accumulation of demands or stressors on mental health was also described when numerous unexpected events would go wrong at work:

Those one-off scenarios, where, it’s gone wrong and it wasn’t expected to go wrong. I think when you’re exposed to them, on a regular basis over a long period of time, it’s very easy to become a bit on edge, all the time, as though you’re waiting for something about to happen. [Participant 2]

This accumulation has caused mental health impacts such as increased anxiety, and the impact was felt to happen without staff members being aware until the mental health damage exists.

#### Individualised responses to stressors

The final theme concerned individual responses to workplace stressors. Participants described strategies such as accepting unavoidable changes (eg, rotas), lowering expectations, and using avoidance coping when dealing with poor management. While these approaches felt helpful, they may partly explain the negative impact of some demands, as they were not always appropriate or sustainable:

It was almost like there’s going to be conflict because there’s a Manager and I’m there. And, it felt like it was really stressful. I felt if a Manager walked into our ambulance station I would go sit in the car or in my, in the ambulance or I would radio control and say I need to go out on standby. [Participant 6]

To further explain how the demands negatively impacted staff, some participants appraised their work situation as a challenge and wanted to learn from it, but others, despite viewing it as a challenge at first, then appraised it as a threat as conditions got worse.

### Why are the identified groups more ‘at risk’ of negative stress-related outcomes?

When exploring why certain staff were more at-risk of negative stress-related outcomes (eg, poor well-being), two subthemes were revealed regarding Band 6s and bank staff (a group not identified in the survey).

#### Band 6 responsibility and support imbalance

Many participants considered Band 6s to experience more work-stress than other Bands due to their extra responsibility, including being responsible for patient discharge, being the lead clinician in an ambulance crew, and having to mentor multiple junior staff:

It felt like we [Band 6] had a lot more weight to carry on our shoulders. I did push back a bit with my line manager, going I don’t mind having this, and he said, well you can apply for a break, but it’s not… I’ve got a student. I want to look after her, and get her through and get her qualified, so I don't want a break. [Participant 4]

In addition to increased responsibility, participants felt support for Band 6s had declined. They previously had time and space to debrief with crews in station hubs, but now felt pressured to work harder and faster. Participants also noted limited career progression for Band 6s and that they were increasingly sought after in other areas (eg, primary care).

#### Negative perceptions of bank staff

Participants suggested bank staff may be at risk partly because some colleagues held unfavourable views of them, believing they benefited from choosing shifts and leaving more night or unsocial shifts to permanent staff. Bank staff felt this perception was unfair and reported receiving inadequate support from the service:

It’s nice being bank, but you’re out on your own a little bit. And initially, I loved it. I’m not loving it anywhere near as much as I used to. Everything’s gone a bit sour, they [The service] just think you turn up and earn a bit of money. But I’ve been in 20 years, so I think I’ve earnt that right to pick and bloody choose a little bit. [Participant 8]

## Discussion

This study used a stress audit to: (1) identify which stressors most negatively impacted ambulance staff’s mental health, well-being and intention to leave, (2) understand why this impact occurred and (3) determine who was most ‘at-risk’. In line with its explanatory sequential design,^32^ the qualitative data were used to extend and deepen interpretation of the quantitative findings rather than to test convergence or divergence between datasets. It concludes that the most harmful stressors for ambulance staff are poor support from upper management, poorly communicated change, excessive organisational demands, and strained workplace relationships, all of which significantly impact mental health, well-being and intention to leave. Importantly, these stressors are not solely the result of frontline pressures; the majority are also deeply rooted in organisational structures and leadership approaches that are compounding staff stress. While line managers provide the daily support and guidance, upper management (eg, workforce directors) sets the organisational tone, develops policies, and ensures resources are available to effectively support staff.^56^ Both levels of management are therefore critical for creating a supportive work environment where staff can thrive. Evidence shows that organisational pressure and management-related stress can be more damaging than traumatic incidents,^9^ and in ambulance services, performance priorities can overshadow people and culture.^57^ More adaptive approaches, such as compassionate leadership, are increasingly advocated^58^ and are associated with lower burnout, greater engagement and improved patient satisfaction.^59^

Management pressure was further reflected in staff experiences of change, which they found demanding due to its sudden implementation, limited supporting evidence, and a top-down approach primarily focused on increasing productivity. Change can reduce staff autonomy by limiting control over workload and decision-making, and as autonomy is a core psychological need,^60^ its loss may diminish motivation and increase burnout risk. Organisational change and its negative impact on NHS staff have been previously identified,^15^ but less so with ambulance staff. Although, making real changes to daily organisational factors that adversely affect ambulance staff is recommended,^17^ caution is required by organisational staff when communicating these changes to ensure well-being of staff alongside service improvement. Similarly, organisational measures intended to improve efficiency, including micromanagement through delivery cells, were associated with increased stress and reduced morale. While the negative impact of rota demands on ambulance staff is well documented,^11^ stress related to delivery cells has not previously been reported, possibly reflecting their novel and potentially hindering nature.^61^ More broadly, ambulance services often prioritise response time targets over training, professional development, and research.^57^

The study highlighted two key explanatory mechanisms, lack of mental rest and sustained threat appraisals, which help explain why stressors have such a detrimental effect on ambulance personnel, with cumulative load further amplifying negative outcomes. Psychological detachment supports recovery and well-being and may buffer work stress^62^; to our knowledge, this is the first study to examine mental rest within the ambulance service. Prior research shows mental rest requires active regulation of cognitive activity and deliberate recovery behaviours,^39^ such as setting boundaries between work and non-work time, reducing rumination and engaging in restorative activities (eg, exercise or quiet reflection). Organisational conditions, including supportive leadership and policies that protect rest and non-work time, can further facilitate detachment.^39 63^ Although ‘mental rest’ is not widely operationalised in other first responder research, it aligns with established concepts such as psychological detachment from work and recovery experiences.^63^ Across police, fire, and other emergency service populations, limited recovery and difficulty disengaging from work have been consistently associated with higher stress, burnout and poorer well-being.^64^

A persistent threat-based appraisal style can harm psychological and physical health^65^ and is a strong predictor of negative outcomes in similar professions, such as midwives.^44^ This study is the first to demonstrate its importance for ambulance staff. Our findings on negative emotions help explain how work stress leads to adverse outcomes: such emotions arise when situations are appraised as threatening or beyond coping capacity.^19^ Accumulated stressors can further shape negative appraisals, reducing perceived coping ability and increasing vulnerability to emotional strain and impaired functioning.^60 65^ For example, after several exhausting shifts, a paramedic may appraise a routine call as overwhelming despite it normally being manageable. One promising approach to lessen threat appraisals, regulate emotions and reduce cumulative load is stress optimisation, which encourages individuals to reinterpret stress and related physiological responses (eg, increased heart or breathing rate) as helpful rather than harmful.^66^

Findings showed that Band 6 front-line paramedics, long-serving staff, male personnel, those working in mixed geographic areas, and bank workers were most at risk. Male staff and those longer in role have previously been identified as vulnerable to burnout in the ambulance sector^18 27^ ; gender-related disparities may reflect variations in coping strategies (eg, greater use of avoidant or suppression-based coping), help-seeking behaviours, and cumulative long-term exposure to occupational stress. In addition, paramedics consistently experience the highest work stress across studies and workforce reports.^27^ Evidence on geographic location is mixed, with some research showing higher anxiety and depression in metropolitan areas,^28^ while others report greater fatigue and distress among rural paramedics.^29^ Band 6 staff were perceived as most stressed due to added responsibilities, such as leading crews, overseeing discharge, and mentoring junior staff. Paramedics often perform clinical tasks similar to hospital doctors but with less formal training and in unpredictable environments requiring autonomous decision-making.^67^ Although bank workers may choose these roles for work-life balance,^68^ our findings suggest they are also at risk, reporting tensions with permanent staff and lower perceived support. Similar issues, including discrimination and reluctance to raise safety concerns, have been reported among NHS temporary staff.^69^ These findings underline the need for equitable organisational support to enable bank staff to contribute effectively to patient care.

Collectively, these findings are building blocks for the structure of a tailored stress management strategy for ambulance staff. The ‘primary’ intervention (aimed at preventing or reducing stressors), which is in line with our findings regarding the impact of the accumulation of stressors, should include both organisational and individual components. At the organisational level, upper management should address system issues (eg, delivery cells, equipment), improve communication, and provide opportunities for staff feedback. They should also protect mandatory breaks and foster a culture that normalises mental rest and detachment. Individual-level components should include education on setting boundaries between work and non-work time, reducing rumination, and engaging in restorative activities. Training in stress optimisation and arousal reappraisal may also help reduce threat appraisals and improve emotion regulation. While there is a plethora of reactive, individual-level interventions (eg, trauma therapy) for ambulance staff,^70^ primary interventions pitched at an organisation-level (eg, job redesign, manager support) are limited.^71^ This is surprising given evidence that primary interventions delivered at both individual and organisational levels benefit staff.^13 15^ Even small organisational changes can improve NHS staff’s working experiences despite the perceived complexity of implementing them.^5^

While this study demonstrates the value of mixed-methods stress audits for understanding work stress and informing targeted interventions,^16^ several limitations should be noted. First, the sample was drawn from a single ambulance service in England, limiting generalisability to other UK or international services, where organisational culture, management practices, and regional demands may differ. Second, the cross-sectional survey design provides only a snapshot of stress at one point in time. Although appropriate for this study’s aims, future work should include a national stress audit to determine whether these findings hold across services and within the broader NHS context. Third, as missing data within the survey were not formally assessed, the possibility of bias due to data not being missing completely at random cannot be excluded. Finally, the intersection of stressors (eg, organisational demands, poor management support) with individual characteristics (eg, gender, Band level) may be more nuanced than presented, indicating the need for further research to better understand these interacting factors.

## Supplementary material

10.1136/bmjopen-2026-119532 online supplemental file 1

## Data Availability

Data are available on reasonable request.
