# Supplementary material for: Work stressors associated with mental health, well-being and intention to leave among ambulance staff in England: an explanatory mixed-methods study
Source: BMJ Open. 2026 Jul 9;16(7):e119532. doi: 10.1136/bmjopen-2026-119532 (PMC13358241; doi:10.1136/bmjopen-2026-119532)
Supplement: online supplemental file 1 [file bmjopen-16-7-s001.docx]

**Supplementary material**

**Interview Prompts: Ambulance Staff**

Context of the interviews: *These interviews will be conducted by telephone or online. The interviews will be conducted with 4 - 6 ambulance staff who are potentially more vulnerable to stress according to the survey data. While this document provides a guide to the questions asked in the interviews, it will likely differ depend on the flow of conversation in each interview. We will also have read the participants survey response prior to interview, and this will prompt some of the more specific questions asked.*

1. Thank, consent and permission to record (record verbal consent if consent form not received)
2. PROMPT WORK BACKGROUND USING SURVEY DATA: age, current role, band level, length of time at Trust, full time/part time/bank, location. What is your previous work experience in emergency care? Have you worked in other locations?

3. INTRODUCE KEY STRESSOR FINDINGS (*Manager support, Relationships, Change and Work demands*) FROM SURVEY AND EXPLORE FOR EACH IN TURN:

- Please describe completely each work stressful experience considering the time, place, location, and factors involved in tensions. Any others? PROMPT: Dimensions of stressors. How often do these situations occur? How long do they typically last? How intense/severe are these demands for you?
- What makes this stressor particularly demanding/intense for you? PROMPT: Properties of stressors. E.g., Novelty, predictability, uncertainty about event, imminence, duration, temporal uncertainty, ambiguity, and timing in relation to life.
- **How** does this demand affect your mental health? Your motivation to stay within or leave the profession? PROMPT: **Why** do you think this is the case?
- Can you describe your thoughts at the time of encountering this stressful experience? How did you evaluate or view this stressor (e.g., a challenge and opportunity for mastery, growth, and development or a threat with possibility of harm)? Do you see this stressor as an opportunity to grow and develop from? How did the stressor make you feel, or did it trigger any particular emotions? Did you feel like you had the resources or ability to cope with this stressor? KEY FOLLOW UP PROMPT TO FOLLOW EACH OF ABOVE QUESTIONS*:* "and what did that mean for/what impact did that have on how you performed, your wellbeing, whether you wanted to stay or leave the profession"?
- What do you feel has been supportive (e.g., personality, social support, workplace culture etc) to help the stressor have less of a negative impact on you? Is there anything within yourself or your environment which has meant the stressor had had a negative impact on you? KEY FOLLOW UP PROMPT TO FOLLOW EACH OF ABOVE QUESTIONS: "and what did that mean for/what impact did that have on how you performed, your wellbeing, whether you wanted to stay or leave the profession"?

3a. MANAGER SUPPORT (*Encouragement and resources provided by the organisation and line management)*: – Are there any aspects related to the support you receive from your organisation and your manager(s) that cause you stress? PROMPT BRIEFLY IF NECESSARY: Do you receive regular and constructive feedback? Do you know what support is available to support you with the emotional demands of your work and how and when to access it? Describe your direct line management support. Does the Trust have policies and procedures to adequately support you? Are systems in place to enable and encourage managers to support you? Are systems in place to enable and encourage staff to support their colleagues? Do you know how to access the required resources to do your job?

3b. RELATIONSHIPS *(Promoting positive working to avoid conflict and dealing with unacceptable behaviour):* Are there any aspects associated with your relationships with colleagues that have caused you stress? PROMPT BRIEFLY IF NECESSARY: Does the Trust promote positive behaviours at work to avoid conflict and ensure fairness? Are systems in place to enable and encourage managers to deal with unacceptable behaviour and/or harassment? Are systems in place to enable and encourage staff to report unacceptable behaviour and/or harassment?

3c. CHANGE: *(Big or small, how it is managed and how it is communicated).* Are there any issues related to organisational change that cause you stress? PROMPT BRIEFLY IF NECESSARY: Does the Trust provide you with timely information to enable you to understand the reasons for proposed changes? Does the Trust ensure adequate consultation on changes and provides opportunities for you to influence proposals? Are you aware of the probable impact of any changes to your job? Are you aware of timetables for changes? Do you have access to relevant support during changes?

3d. WORK DEMANDS (*workload, work patterns and the work environment):* What are the key work demands that cause you stress? PROMPT BRIEFLY IF NECESSARY: Does the Trust provide you with adequate and achievable demands in relation to the agreed hours of work? Is there pressure to work long hours? Are there unrealistic time pressures? Are your skills and abilities are matched to the job demands? Is your role designed to be within your capabilities? Do you have specific concerns about your work environment? Are these addressed?

4. EXPLORE MENTAL REST

Good mental rest results in ‘feeling fresh’ valuing your job’ and being able to apply lots of effort in your job’. Level of mental rest is determined by how much you can switch off from work and how well you are sleeping. Do you think you get adequate mental rest (AT WORK, AT HOME)? If not, why not?

How does not getting mental rest affect you at work? How does getting adequate mental benefit you at work? What about its impact on your wellbeing and mental health – how does it impact this and why? What about its impact on your intention to leave or stay in the profession – how does it impact this and why? PROMPTS: Clearer thinking? More relaxed and positive emotions, reduced negative emotions? More adaptive behaviours? Better physical health?

5. AT RISK GROUPS

The 3 most vulnerable groups revealed from the survey are those who have had more time in their role, Band 6s and those who are working in mixed areas (vs just urban areas). In relation to your own experiences, why do you feel that more experienced/Band 6/mixed area employees reported poorer outcomes (such as a greater intention to leave/poorer work satisfaction) etc? What might be the reasons for this?

Other vulnerable groups included Frontline ambulance staff and males. Why do you these groups report poorer outcomes? – how might you explain that?

6. Is there anything else you would like to add?

*Thank you and end. Remind all participants of support resources in participant information sheet*

**Supplementary Table 1.** Means, standard deviations, and intercorrelations of stress-related variables and outcomes.

|  | *M* | *SD* | 1.  D | 2.  WB | 3.  ITL | 4.  DE | 5.  CO | 6.  MS | 7.  PS | 8.  RE | 9.  RO | 10. CH | 11. SA |
| --- | --- | --- | --- | --- | --- | --- | --- | --- | --- | --- | --- | --- | --- |
| 1. D | 2.39 | 1.69 |  |  |  |  |  |  |  |  |  |  |  |
| 2. WB | 39.94 | 21.65 | **-0.51** |  |  |  |  |  |  |  |  |  |  |
| 3. ITL | 2.97 | 1.30 | **0.45** | **-0.42** |  |  |  |  |  |  |  |  |  |
| 4. DE | 2.83 | 0.83 | **-0.43** | **0.37** | **-0.49** |  |  |  |  |  |  |  |  |
| 5. CO | 2.89 | 0.89 | **-0.23** | **0.38** | **-0.35** | **0.34** |  |  |  |  |  |  |  |
| 6. MS | 3.04 | 0.96 | **-0.32** | **0.41** | **-0.48** | **0.39** | **0.51** |  |  |  |  |  |  |
| 7. PS | 3.67 | 0.73 | **-0.26** | **0.35** | **-0.37** | **0.23** | **0.25** | **0.51** |  |  |  |  |  |
| 8. RE | 3.78 | 0.93 | **-0.36** | **0.16** | **-0.47** | **0.48** | **0.18** | **0.29** | **0.42** |  |  |  |  |
| 9.. RO | 3.68 | 0.84 | **-0.24** | **0.30** | **-0.39** | **0.30** | **0.46** | **0.46** | **0.44** | **0.33** |  |  |  |
| 10. CH | 2.61 | 0.91 | **-0.25** | **0.42** | **-0.42** | **0.39** | **0.62** | **0.71** | **0.26** | **0.12** | **0.44** |  |  |
| 11. SA | -0.37 | 1.67 | **-0.40** | **0.47** | **-0.29** | **0.45** | **0.30** | **0.22** | **0.21** | **0.19** | **0.29** | **0.21** |  |
| 12. MR | 2.51 | 1.20 | **-0.42** | **0.63** | **-0.43** | **0.42** | **0.43** | **0.42** | **0.18** | **0.14** | **0.26** | **0.52** | **0.43** |

***Notes:*** Values in bold are significant at *p* < .05

Higher scores on each subscale on the HSE-MS IT Tool reflect better standards and thus lower work-related stress

WHO-5 scores (for Welling [WB]) were calculated by summing the five items (0–5 each; total raw score range 0–25) and multiplying by 4 to obtain a percentage score ranging from 0 to 100, with higher scores indicating better wellbeing;

D = Depression; WB = Wellbeing; Satisfaction; ITL = Intention to Leave; DE = HSE Demands; CO = HSE Control; MS = HSE Manager Support; PS = HSE Peer Support; RE = HSE Relationships; RO = HSE Role; CH = HSE Change; SA = Stress Appraisal; MR = Mental Rest.

**Supplementary Table 2.** Mean and standard deviations for work and demographic characteristics for outcome variables.

|  | **Depression** | | **Wellbeing** | | **Intention to leave** | |
| --- | --- | --- | --- | --- | --- | --- |
|  | *M* | *SD* | *M* | *SD* | *M* | *SD* |
| **Gender** |  |  |  |  |  |  |
| Female | 2.19 | 1.68 | 40.43 | 20.69 | 2.69 | 1.25 |
| Male | 2.57 | 1.70 | 39.72 | 22.66 | 3.14 | 1.32 |
| **Age** |  |  |  |  |  |  |
| 18 – 24 years | 2.95 | 1.32 | 38.67 | 13.88 | 2.76 | 1.00 |
| 25 – 34 years | 2.23 | 1.51 | 42.45 | 20.66 | 2.92 | 1.26 |
| 35 – 44 years | 2.33 | 1.78 | 39.56 | 24.02 | 2.90 | 1.34 |
| 45 – 54 years | 2.61 | 1.87 | 35.96 | 21.97 | 3.04 | 1.43 |
| 55 + years | 2.25 | 1.78 | 42.57 | 22.00 | 3.04 | 1.32 |
| **Location** |  |  |  |  |  |  |
| A rural area | 2.45 | 1.89 | 39.13 | 23.50 | 3.00 | 1.33 |
| An urban area | 2.36 | 1.56 | 44.37 | 20.72 | 2.76 | 1.21 |
| Mixed - rural and urban | 2.40 | 1.67 | 36.83 | 20.78 | 3.09 | 1.38 |
| **Experience** |  |  |  |  |  |  |
| Less than 2 years | 1.81 | 1.43 | 41.33 | 20.11 | 2.25 | 1.10 |
| 2 - 5 years | 2.33 | 1.84 | 42.00 | 20.52 | 2.81 | 1.32 |
| 5 - 10 years | 2.55 | 1.38 | 40.62 | 21.82 | 3.21 | 1.18 |
| More than 10 years | 2.78 | 1.91 | 35.69 | 23.26 | 3.31 | 1.39 |
| **Band** |  |  |  |  |  |  |
| Band 3 or below | 2.05 | 1.74 | 41.86 | 22.24 | 2.46 | 1.37 |
| Band 4 | 2.60 | 1.61 | 41.83 | 21.35 | 2.40 | 1.14 |
| Band 5 | 2.42 | 1.39 | 41.52 | 21.49 | 3.03 | 1.07 |
| Band 6 | 2.56 | 1.84 | 35.66 | 21.20 | 3.43 | 1.33 |
| Band 7 | 2.16 | 1.39 | 43.75 | 20.32 | 2.87 | 1.10 |
| Band 8 and above | 3.00 | 1.95 | 46.67 | 21.87 | 2.83 | 1.27 |
| **Role type** |  |  |  |  |  |  |
| Frontline ambulance | 2.36 | 1.77 | 37.73 | 21.79 | 3.01 | 1.36 |
| Clinical Hubs and Dispatch | 2.77 | 1.32 | 44.56 | 19.53 | 3.11 | 1.08 |
| Support roles | 2.15 | 1.42 | 46.70 | 21.99 | 2.55 | 1.20 |
| **Work capacity** |  |  |  |  |  |  |
| Full time | 2.35 | 1.68 | 40.39 | 22.26 | 2.89 | 1.27 |
| Part time | 2.53 | 1.63 | 37.75 | 17.70 | 3.21 | 1.47 |
| Bank | 2.86 | 1.92 | 37.14 | 22.38 | 3.43 | 1.45 |

**Supplementary Table 3.** Mean and standard deviations for work and demographic characteristics for stress-related variables.

|  | **Demands** | | **Control** | | **Manager support** | | **Peer support** | | **Relationships** | | **Role** | | **Change** | | **Stress appraisal** | | **Mental Rest** | |
| --- | --- | --- | --- | --- | --- | --- | --- | --- | --- | --- | --- | --- | --- | --- | --- | --- | --- | --- |
|  | *M* | *SD* | *M* | *SD* | *M* | *SD* | *M* | *SD* | *M* | *SD* | *M* | *SD* | *M* | *SD* | *M* | *SD* | *M* | *SD* |
| **Gender** |  |  |  |  |  |  |  |  |  |  |  |  |  |  |  |  |  |  |
| Female | 2.95 | 0.78 | 2.94 | 0.85 | 3.24 | 0.94 | 3.77 | 0.71 | 3.91 | 0.91 | 3.86 | 0.77 | 2.78 | 0.85 | -0.42 | 1.67 | 2.58 | 1.15 |
| Male | 2.73 | 0.82 | 2.89 | 0.93 | 2.93 | 0.93 | 3.62 | 0.73 | 3.69 | 0.94 | 3.57 | 0.86 | 2.52 | 0.93 | -0.25 | 1.64 | 2.51 | 1.27 |
| **Age** |  |  |  |  |  |  |  |  |  |  |  |  |  |  |  |  |  |  |
| 18 – 24 years | 2.95 | 0.62 | 2.98 | 0.79 | 2.87 | 0.92 | 3.54 | 0.76 | 3.60 | 0.70 | 3.60 | 0.76 | 2.67 | 1.01 | -0.43 | 1.72 | 2.57 | 1.17 |
| 25 – 34 years | 2.80 | 0.80 | 3.03 | 0.81 | 3.17 | 0.85 | 3.74 | 0.66 | 3.68 | 0.98 | 3.70 | 0.71 | 2.80 | 0.90 | -0.21 | 1.41 | 2.78 | 1.15 |
| 35 – 44 years | 2.80 | 0.75 | 2.84 | 0.87 | 3.07 | 0.97 | 3.57 | 0.82 | 3.77 | 0.89 | 3.63 | 0.90 | 2.61 | 0.89 | -0.63 | 1.76 | 2.44 | 1.26 |
| 45 – 54 years | 2.87 | 0.93 | 2.79 | 1.02 | 2.98 | 1.08 | 3.77 | 0.73 | 3.93 | 0.95 | 3.78 | 0.98 | 2.41 | 0.93 | -0.11 | 1.90 | 2.21 | 1.21 |
| 55 + years | 2.78 | 0.74 | 2.85 | 0.91 | 2.88 | 1.00 | 3.58 | 0.75 | 4.11 | 0.86 | 3.74 | 0.71 | 2.46 | 0.68 | -0.71 | 1.36 | 2.61 | 1.20 |
| **Location** |  |  |  |  |  |  |  |  |  |  |  |  |  |  |  |  |  |  |
| A rural area | 2.86 | 0.90 | 2.85 | 0.90 | 3.02 | 1.00 | 3.72 | 0.81 | 3.92 | 0.87 | 3.73 | 0.81 | 2.59 | 0.81 | -0.28 | 1.76 | 2.59 | 1.30 |
| An urban area | 2.98 | 0.78 | 3.13 | 0.90 | 3.30 | 0.84 | 3.65 | 0.65 | 3.66 | 1.00 | 3.76 | 0.83 | 3.02 | 0.92 | -0.17 | 1.39 | 2.84 | 1.14 |
| Mixed | 2.69 | 0.77 | 2.75 | 0.86 | 2.86 | 1.00 | 3.68 | 0.76 | 3.80 | 0.92 | 3.62 | 0.85 | 2.34 | 0.84 | -0.51 | 1.79 | 2.27 | 1.16 |
| **Experience** |  |  |  |  |  |  |  |  |  |  |  |  |  |  |  |  |  |  |
| < 2 years | 3.14 | 0.72 | 3.02 | 0.79 | 3.38 | 0.90 | 3.95 | 0.65 | 4.12 | 0.85 | 3.82 | 0.81 | 2.78 | 0.80 | -0.18 | 1.49 | 2.64 | 1.09 |
| 2 - 5 years | 2.91 | 0.98 | 2.70 | 0.85 | 3.06 | 1.03 | 3.84 | 0.71 | 3.91 | 0.89 | 3.72 | 0.80 | 2.51 | 0.90 | -0.09 | 1.78 | 2.44 | 1.27 |
| 5 - 10 years | 2.65 | 0.66 | 3.06 | 0.87 | 3.10 | 0.85 | 3.52 | 0.67 | 3.47 | 0.93 | 3.58 | 0.82 | 2.81 | 0.95 | -0.36 | 1.37 | 2.72 | 1.23 |
| >10 years | 2.71 | 0.80 | 2.76 | 0.96 | 2.71 | 0.97 | 3.51 | 0.80 | 3.76 | 0.94 | 3.65 | 0.91 | 2.33 | 0.86 | -0.56 | 1.95 | 2.31 | 1.23 |
| **Band** |  |  |  |  |  |  |  |  |  |  |  |  |  |  |  |  |  |  |
| Band 3 or below | 2.95 | 0.86 | 2.56 | 0.91 | 3.22 | 1.07 | 3.85 | 0.67 | 3.94 | 0.93 | 3.68 | 0.95 | 2.56 | 0.94 | 0.00 | 1.74 | 2.51 | 1.24 |
| Band 4 | 3.08 | 0.63 | 3.27 | 0.80 | 3.24 | 0.76 | 3.59 | 0.62 | 3.59 | 0.93 | 3.79 | 0.90 | 3.19 | 0.86 | -0.31 | 1.23 | 3.20 | 1.05 |
| Band 5 | 2.73 | 0.75 | 3.04 | 0.79 | 3.02 | 0.91 | 3.53 | 0.81 | 3.54 | 0.94 | 3.68 | 0.81 | 2.73 | 0.93 | -0.52 | 1.32 | 2.63 | 1.11 |
| Band 6 | 2.70 | 0.83 | 2.74 | 0.86 | 2.73 | 0.96 | 3.71 | 0.77 | 3.87 | 0.91 | 3.66 | 0.80 | 2.26 | 0.79 | -0.53 | 1.90 | 2.15 | 1.18 |
| Band 7 | 2.90 | 0.85 | 3.51 | 0.64 | 3.42 | 0.74 | 3.66 | 0.59 | 3.84 | 0.91 | 3.82 | 0.62 | 2.99 | 0.70 | -0.06 | 1.54 | 2.75 | 1.19 |
| Band 8 and above | 2.92 | 0.54 | 3.50 | 0.80 | 3.62 | 0.57 | 3.48 | 0.67 | 3.79 | 1.01 | 3.78 | 0.72 | 3.39 | 0.55 | -0.75 | 1.66 | 3.08 | 1.24 |
| **Role type** |  |  |  |  |  |  |  |  |  |  |  |  |  |  |  |  |  |  |
| Frontline ambulance | 2.71 | 0.81 | 2.71 | 0.86 | 2.88 | 0.98 | 3.75 | 0.72 | 3.83 | 0.91 | 3.66 | 0.84 | 2.38 | 0.87 | -0.37 | 1.78 | 2.35 | 1.21 |
| CHD | 2.86 | 0.64 | 3.05 | 0.87 | 3.23 | 0.73 | 3.46 | 0.58 | 3.21 | 0.90 | 3.68 | 0.87 | 3.09 | 0.80 | -0.21 | 1.31 | 3.07 | 1.12 |
| Support roles | 3.25 | 0.72 | 3.54 | 0.72 | 3.49 | 0.97 | 3.55 | 0.90 | 4.08 | 0.81 | 3.83 | 0.85 | 3.23 | 0.74 | -0.30 | 1.22 | 2.93 | 1.10 |
| **Work capacity** |  |  |  |  |  |  |  |  |  |  |  |  |  |  |  |  |  |  |
| Full time | 2.83 | 0.81 | 2.92 | 0.87 | 3.12 | 0.94 | 3.68 | 0.71 | 3.75 | 0.92 | 3.67 | 0.83 | 2.69 | 0.90 | -0.26 | 1.59 | 2.58 | 1.26 |
| Part time | 2.82 | 0.82 | 2.96 | 0.95 | 2.87 | 1.02 | 3.75 | 0.83 | 3.98 | 1.01 | 3.86 | 0.82 | 2.36 | 0.89 | -0.61 | 1.88 | 2.30 | 0.96 |
| Bank | 2.66 | 0.81 | 2.25 | 0.98 | 2.20 | 0.90 | 3.43 | 0.77 | 3.61 | 0.98 | 3.45 | 0.94 | 2.19 | 0.82 | -1.00 | 1.92 | 2.50 | 1.09 |

*Note.* CHD = Clinical Hubs and Dispatch; Higher scores on each subscale on the HSE-MS IT Tool reflect better standards and thus lower work-related stress
