# Supplementary material for: Work stressors associated with mental health, well-being and intention to leave among ambulance staff in England: an explanatory mixed-methods study
Source: BMJ Open. 2026 Jul 9;16(7):e119532. doi: 10.1136/bmjopen-2026-119532 (PMC13358241; doi:10.1136/bmjopen-2026-119532)
Supplement: Supplementary file 2 [file bmjopen-2026-119532.reviewer_comments.pdf]

PEER REVIEW HISTORY

BMJ Open publishes all reviews undertaken for accepted manuscripts. Reviewers are asked to complete a checklist review form and are provided with free text boxes to elaborate on their assessment. These free text comments are reproduced below.

ARTICLE DETAILS

Title (Provisional)

Work stressors associated with mental health, wellbeing and intention to leave among ambulance staff in England: an explanatory mixed-methods study

Authors

Anchors, Zoe; Arnold, Rachel; Moore, Lee; Nicholson, Helen; Voss, Sarah; Walsh, Nicola E; Black, Sarah

---

VERSION 1 - REVIEW

**Reviewer**	**1**
**Name**	**Baek, Eun-Mi**
**Affiliation**	**The Catholic University of Korea, Department of Preventive Medicine**
**Date**	**15-Apr-2026**
**COI**	**None**

Comment 1: Missing data handling not described in Methods

The authors report considerable proportions of missing data across several key demographic and work-related variables (e.g., sex: 17.1%; primary work location: 15.0%; role type: 15.0%; length of time in role: 16.9%; Band level: 16.0%). However, the Methods section provides no description of how missing data were handled in the analyses. It is unclear whether listwise deletion, pairwise deletion, or any form of imputation was applied. Given that the proportion of missing data is non-trivial and could introduce systematic bias — particularly if data are not missing completely at random (MCAR) — this omission is a significant methodological concern.

Recommendation: The authors should explicitly state the missing data handling strategy in the Data Analysis subsection. Where appropriate, a missing data analysis (e.g., Little's MCAR test, or comparison of responders vs. non-responders on available variables) should be reported or at minimum discussed as a limitation.

Comment 2: Single-item measure for intention to leave lacks justification

Intention to leave — one of the three primary outcome variables in this study — is assessed using a single item ("I want to leave the ambulance service") rated on a 5-point scale. The use of a single-item measure for a key outcome raises serious concerns regarding construct validity and measurement reliability, as internal consistency (e.g., Cronbach's alpha) cannot be computed for a single item and test-retest reliability is not reported. Notably, while the authors provide reliability coefficients for all multi-item scales (e.g., WHO-5: $\alpha = .91$; HSE subscales), no psychometric justification is offered for this single-item measure. The Methods section contains no citation supporting the validity of this approach, nor any acknowledgement of its limitations.

The authors must provide explicit justification for the use of a single-item measure. This should include: (a) a citation of prior studies that have validated or employed this item in comparable occupational health research, and (b) acknowledgement of the psychometric limitations of single-item measurement, either in the Methods or Limitations section.

Comment 3: Insufficient justification for sample size and data saturation in qualitative component

The qualitative component involved interviews with only eight participants (seven ambulance staff and one occupational health professional). While the authors note that qualitative data were analysed using thematic analysis, no justification is provided for why eight interviews were considered sufficient to generate credible and well-developed themes. Specifically, the manuscript does not address whether data saturation or informational redundancy was reached, which is a standard criterion for evaluating the adequacy of qualitative sample sizes in thematic analysis. Furthermore, the inclusion of an occupational health professional whose role and perspective differ substantially from those of ambulance staff is not addressed analytically. It is unclear whether data from this participant were analysed separately or integrated with staff data, and how this may have influenced theme development.

The authors should: (a) provide an explicit rationale for the sample size of eight, with reference to relevant literature on sample size adequacy in thematic analysis (e.g., Braun & Clarke, 2021; Fugard & Potts, 2015); (b) state clearly whether data saturation was assessed and at what point it was considered reached; and (c) clarify how data from the occupational health professional were treated in the analysis and justify their inclusion in a study focused on ambulance staff stress experiences.

Comment 4: Reference 4 relies on a non-peer-reviewed, grey literature source of low academic credibility

Reference 4, cited to support the statement that "staff leaving ambulance services has risen by 51% since 2019," directs readers to a Google Sheets document published by the Liberal Democrats (a UK political party). This source has not undergone peer review, cannot be verified for methodological rigour, and represents politically motivated grey literature. Relying on a political party's spreadsheet to substantiate a key epidemiological claim in the Introduction is inappropriate for a submission to an academic journal and risks undermining the credibility of the manuscript.

This reference should be replaced with a peer-reviewed publication or an official institutional data source (e.g., NHS Digital, NHS England workforce statistics, or the Health and Social Care Information Centre). If no peer-reviewed source is available, the claim should be reframed accordingly and the source described transparently, with its limitations acknowledged.

Comment 5: Integration of quantitative and qualitative findings is insufficiently developed

The manuscript presents quantitative and qualitative findings in separate sections and largely uses the qualitative data to confirm or illustrate the survey results. While this is consistent with an explanatory sequential design, the Discussion does not provide an explicit and systematic integration of the two datasets. Specifically, the authors do not address: (a) the extent to which the qualitative findings *converge with* versus *expand upon* the quantitative results; (b) any instances of divergence between the two data sources and how these are interpreted; and (c) what the combined findings offer that neither dataset could provide alone. This limits the added value of the mixed-methods approach and does not fully meet the standards expected for mixed-methods research reporting (e.g., O'Cathain et al., 2010).

The Discussion should include a dedicated integrative paragraph that explicitly addresses convergence, expansion, and any divergence between the quantitative and qualitative findings, and articulates the contribution of the mixed-methods design to the study's conclusions.

Comment 6: Placement of Figure 1 disrupts manuscript flow

Figure 1, which maps survey findings to interview themes across the three research questions, is positioned within the Qualitative Findings section, following the first two thematic subheadings. As this figure is intended to provide an integrative overview of the entire thematic structure, its current placement mid-section and after partial presentation of themes means readers

encounter it before having access to the full thematic content it summarises. This disrupts comprehension and reduces the figure's orienting function.

Figure 1 should be relocated to immediately precede the qualitative thematic findings section, so that it serves as a structural map before the themes are described in detail. Alternatively, if the journal format requires figures at the end, the in-text reference should clearly direct readers to consult the figure before reading the thematic descriptions.

**Reviewer**	**2**
**Name**	**Mielke, John**
**Affiliation**	**University of Waterloo, School of Public Health Sciences**
**Date**	**17-Apr-2026**
**COI**	**None**

Abstract

The abstract is clearly written and provides a good overview of the project and its results. One suggestion, though, would be to edit the Methods section of the abstract such that the ‘stress elements’ and the ‘key outcomes’ are phrased in the same manner as in the main document. That is, instead of ‘stressors’ and ‘appraisals’, ’work stressors’ and ‘stress appraisals’ should be used. Similarly, instead of ‘mental health’, ‘depression’ should be used.

Introduction

The Introduction does a splendid job of clearly and succinctly explaining why the authors set out to perform a stress audit within ambulance staff. Two suggestions, however, are proposed:

a) On line 28, there appears to be a typographical error in how the citation is presented to support the noted point.

b) In the final paragraph, the authors note that the stress audit was informed by two frameworks; although the first one of these (transactional stress theory) is described in an earlier paragraph, the same is not true of the second one (transdisciplinary model of stress). As a result, the authors should devote a few lines to informing the reader about the transdisciplinary model of stress and its relationship to the study.

Materials and Methods

a) On lines 10-12, the authors note that their survey was completed by 420 staff; a nice addition would be to inform the reader what proportion of the overall staff at this particular service is represented by those who participated.

b) In Table 1, the authors note that they had 420 participants, yet when the number of participants in the 'sex' category are summed the result is 412.

c) Carrying on with Table 1, the authors should consider changing 'sex' to 'gender', which is how they refer to this parameter through-out the rest of the manuscript.

d) As well, in Table 1, the authors include a row for each category labelled 'missing', but its meaning is unclear. That is, are those labelled as 'missing' individuals on staff who chose not to participate (assuming that the total staff numbered 420), or are they those participants who did not enter a particular piece of information?

e) As a final point about Table 1, the authors should define, or explain what 'bank' means within this context, for the benefit of international readers. Similarly, 'GDPR' (line 160 should be defined.

f) To help the reader appreciate a context for the results from this sample of ambulance staff, the authors should consider providing examples of the values that have been observed in the 'general population'. For instance, the average PHQ-2 score reported by the authors for their sample was 2.39 and it would be informative to see how this compares to what has been found in the broader population.

g) Since Cohen's d is typically used as an effect size measure when considering the difference between two means, the authors may want to either explain, or include a citation to expand upon the application of Cohen's d to a linear regression model.

Results

a) In Table 3, the mean for the wellbeing scale is presented as '39.94', yet the authors defined the scale as having 5 items, each with a maximum value of 5 (meaning that the maximum score would be expected to be 25). Please clarify.

b) Similarly, the mean values presented for the demographic parameters do not seem appropriate. For example, the mean value for age is '2.92' and for gender is '1.54'.

c) With regards to Table 4, could the authors provide a brief explanation for their decision to include both the R2 and Cohen's d as measures of effect size? Also, were steps taken to determine whether bias existed in their residuals?

d) For Table 5, would the authors consider including an effect size measure to allow these data to be presented in a manner consisted with those found in other tables?

e) In Table 6, there are 346 degrees of freedom stated, which would allow a reader to assume that the sample size was 348 (given that an independent t-test was used). However, the authors report in Table 1 that there were 160 female participants and 180 male participants, which sum to a sample size of 340. Please clarify.

f) Whilst lines 51-52 state that 'stress appraisal predicted wellbeing only', this does not seem to align with Table 4, which shows that stress appraisal predicts depression, wellbeing, and intention to leave scores. Please clarify.

g) Certain items in the 'qualitative findings' section could be explained for the benefit of readers from outside the UK. In particular, the meaning of a 'delivery cell', 'rota', and 'bank staff'.

h) A small point, but the font size and clarity in Figure 1 could be enhanced to improve readability.

i) On page 18, the quotation highlighted in lines 37-44 nicely illustrates the point about suppressing emotions, however, the authors could more clearly connect the quotation to the preceding paragraph, which addresses the suppressing of emotions relating to stressors such as end-of-shift demands. In other words, the reader is presented with a quotation that clearly touches on stress caused by emotional suppression, but the quotation does not explain what caused the anger and frustration that the participant had to lock away.

Discussion

a) On lines 49-51, (page 21) the authors state 'making real changes to daily organisational factors that impact ambulance staff is recommended'. Given the phrasing of the sentence, the reader can presume the authors intended that the 'impact' was positive, however, an 'impact' is not always positive. As a result, the authors may wish to edit the passage for clarity.

b) The discussion around 'mental rest' was interesting, and I wonder whether the authors would consider expanding the section to comment on what is known (if anything) about the perception and importance of 'mental rest' amongst other first responders (police and those in the fire service).

c) Arguably, the most important sociodemographic variable is sex/gender. Given that the authors observed differences related to sex/gender, the Discussion section would be enhanced if they were to touch on these findings a bit more. For example, why might male staff be more at risk?

VERSION 1 - AUTHOR RESPONSE

Thank you very much for all of your insightful comments. We have made your suggested changes and feel they have further strengthened the manuscript. We have included our responses to each comment below and indicated using page numbers where highlighted revisions to the manuscript (marked copy) can be viewed (via track-changes).

Reviewer	Comment Number	Location	Comment	Action
Reviewer 1	1	Methods	Missing data handling not described in Methods.<br>The authors report considerable proportions of missing data across several key demographic and work-	We have clarified the handling of missing data in the Methods section (See Page 7). Analyses were conducted using all available data for each

			related variables (e.g., sex: 17.1%; primary work location: 15.0%; role type: 15.0%; length of time in role: 16.9%; Band level: 16.0%). However, the Methods section provides no description of how missing data were handled in the analyses. It is unclear whether listwise deletion, pairwise deletion, or any form of imputation was applied. Given that the proportion of missing data is non-trivial and could introduce systematic bias — particularly if data are not missing completely at random (MCAR) — this omission is a significant methodological concern. Recommendation: The authors should explicitly state the missing data handling strategy in the Data Analysis subsection. Where appropriate, a missing data analysis (e.g., Little's MCAR test, or comparison of responders vs. non-responders on available variables) should be reported or at minimum discussed as a limitation.	variable, with denominators varying due to missingness.<br><br>We have also acknowledged the potential implications of missing data in the Limitations section (See Page 25).<br>Even with missing data, we consider our analyses to be sufficiently powered (e.g., Schonbrodt & Perugini, 2013).
	2	Methods	Single-item measure for intention to leave lacks justification<br>Intention to leave — one of the three primary outcome variables in this study — is assessed using a single item ("I want to leave the ambulance service") rated on a 5-point scale. The use of a single-item measure for a key outcome raises serious concerns regarding construct validity and measurement reliability, as internal consistency (e.g., Cronbach's	We acknowledge that intention to leave was assessed using a single-item measure ("I want to leave the ambulance service"), and we agree that single-item measures do not allow for the assessment of internal consistency reliability (e.g., Cronbach's alpha). Our decision to use a single-item indicator was guided by both conceptual and practical considerations.

			alpha) cannot be computed for a single item and test-retest reliability is not reported. Notably, while the authors provide reliability coefficients for all multi-item scales (e.g., WHO-5: α = .91; HSE subscales), no psychometric justification is offered for this single-item measure. The Methods section contains no citation supporting the validity of this approach, nor any acknowledgement of its limitations.<br>The authors must provide explicit justification for the use of a single-item measure. This should include: (a) a citation of prior studies that have validated or employed this item in comparable occupational health research, and (b) acknowledgement of the psychometric limitations of single-item measurement, either in the Methods or Limitations section.	First, intention to leave is widely regarded as a relatively concrete, unidimensional construct that can be meaningfully captured using a direct question. Recent methodological literature has noted that single-item measures may be appropriate where constructs are straightforward and face-valid, particularly in occupational and organisational research (Allen et al., 2022). Prior research in occupational stress with healthcare professionals has employed a single-item measures of turnover intention, particularly in applied settings where survey brevity is essential. For example, Anchors et al. (2024) used this single item measure when investigating occupational stress amongst midwives. Further, studies such as Meese et al. (2024) demonstrated that single-item measures of job attitudes and withdrawal cognitions can show acceptable validity and strong correlations with multi-item scales.<br><br>Second, the use of a single-item measure helped reduce respondent burden in a survey that already included multiple validated multi-item instruments which were also brief (e.g., WHO-5, HSE-MS IT, PHQ-2) given the time-constrained and high-demand context of ambulance personnel.<br><br>We have now revised the Methods section to include appropriate citations supporting the use of single-item measures for intention to

				leave and have explicitly acknowledged the limitations of this approach (See Pages 6-7).
	3	Results	Insufficient justification for sample size and data saturation in qualitative component.<br>The qualitative component involved interviews with only eight participants (seven ambulance staff and one occupational health professional). While the authors note that qualitative data were analysed using thematic analysis, no justification is provided for why eight interviews were considered sufficient to generate credible and well-developed themes. Specifically, the manuscript does not address whether data saturation or informational redundancy was reached, which is a standard criterion for evaluating the adequacy of qualitative sample sizes in thematic analysis. Furthermore, the inclusion of an occupational health professional whose role and perspective differ substantially from those of ambulance staff is not addressed analytically. It is unclear whether data from this participant were analysed separately or integrated with staff data, and how this may have influenced theme development.<br>The authors should: (a) provide an explicit rationale for the sample size of eight, with reference to relevant literature on sample size	First, regarding sample size, our qualitative component was designed to provide in-depth, contextual insight to complement the quantitative findings rather than to achieve exhaustive thematic coverage. The sample size of eight interviews is consistent with guidance for reflexive thematic analysis, where smaller samples can be appropriate when the aim is to generate rich, detailed accounts rather than broad generalisability. As noted by Braun & Clarke (2021), thematic analysis does not require strict adherence to data saturation as a criterion, and sample size adequacy should instead be judged in relation to the study's analytic purpose and depth of engagement with the data. In addition, models of thematic sufficiency (e.g., Fugard & Potts, 2015) suggest that relatively small samples may be sufficient when the scope of themes is focused and the participant group is relatively homogeneous. Our sample can be considered relatively homogeneous, as all participants were drawn from the same organisation and, consistent with the quantitative findings, reported comparably high levels of key workplace stressors such as work demands.<br><br>Second, regarding saturation, we acknowledge that we did not explicitly report this in the original manuscript. Consistent

			adequacy in thematic analysis (e.g., Braun & Clarke, 2021; Fugard & Potts, 2015); (b) state clearly whether data saturation was assessed and at what point it was considered reached; and (c) clarify how data from the occupational health professional were treated in the analysis and justify their inclusion in a study focused on ambulance staff stress experiences.	with reflexive thematic analysis, we did not treat saturation as a formal stopping criterion. However, during analysis we observed a high degree of informational redundancy across interviews, with no substantially new themes generated from later interviews.<br>We have now clarified these issues in the Methods section and explicitly stated our analytic approach to saturation (see Page 9), in line with contemporary qualitative guidance (Fugard & Potts, 2015; Braun & Clarke, 2021; Hennink, Kaiser, & Marconi, 2017).<br><br>Third, regarding the inclusion of the occupational health professional, we agree that this requires clarification. The occupational health participant was included to provide a complementary organisational perspective on staff wellbeing and retention. Their data were analysed alongside ambulance staff data within the same thematic framework but were used primarily to contextualise and triangulate staff accounts rather than to drive theme generation.<br><br>We have revised the manuscript accordingly to (a) provide a clear rationale for the sample size with reference to relevant qualitative methodology literature (see Page 9), (b) clarify our approach to saturation and analytic sufficiency (see Page 9), and (c) explain how the occupational health interview

				was incorporated into the analysis (see Page 9).
	4	Introduction	Reference 4 relies on a non-peer-reviewed, grey literature source of low academic credibility<br>Reference 4, cited to support the statement that "staff leaving ambulance services has risen by 51% since 2019," directs readers to a Google Sheets document published by the Liberal Democrats (a UK political party). This source has not undergone peer review, cannot be verified for methodological rigour, and represents politically motivated grey literature. Relying on a political party's spreadsheet to substantiate a key epidemiological claim in the Introduction is inappropriate for a submission to an academic journal and risks undermining the credibility of the manuscript.<br>This reference should be replaced with a peer-reviewed publication or an official institutional data source (e.g., NHS Digital, NHS England workforce statistics, or the Health and Social Care Information Centre). If no peer-reviewed source is available, the claim should be reframed accordingly and the source described transparently, with its limitations acknowledged.	We agree that the originally cited source does not represent an appropriate academic reference due to its lack of peer review and limited transparency regarding data provenance. We acknowledge that this may have undermined the perceived robustness of the statement as originally presented.<br><br>In response, we have removed the reference and replaced it with official workforce statistics from NHS England which provide systematically collected and publicly reported data on ambulance service staffing trends. We have also revised the wording of the statement to ensure it accurately reflects the available national workforce data (see Page 2).
	5	Discussion	Integration of quantitative and qualitative findings is insufficiently developed.<br>The manuscript presents quantitative and qualitative findings in separate sections and largely uses the	We agree that clearer articulation of how the two datasets relate enhances the coherence and interpretability of mixed-methods research.<br>However, we would like to clarify that this study followed

			qualitative data to confirm or illustrate the survey results. While this is consistent with an explanatory sequential design, the Discussion does not provide an explicit and systematic integration of the two datasets. Specifically, the authors do not address: (a) the extent to which the qualitative findings converge with versus expand upon the quantitative results; (b) any instances of divergence between the two data sources and how these are interpreted; and (c) what the combined findings offer that neither dataset could provide alone. This limits the added value of the mixed-methods approach and does not fully meet the standards expected for mixed-methods research reporting (e.g., O'Cathain et al., 2010).<br>The Discussion should include a dedicated integrative paragraph that explicitly addresses convergence, expansion, and any divergence between the quantitative and qualitative findings, and articulates the contribution of the mixed-methods design to the study's conclusions.	an explanatory sequential mixed-methods design, in which the qualitative component was specifically used to elaborate and provide contextual understanding of the quantitative findings (Creswell & Creswell, 2017), rather than to formally test convergence or divergence between datasets. As such, the qualitative findings were intended to explain and extend the survey results rather than to independently corroborate or contradict them.<br><br>In line with this design, we have now added an integrative paragraph at the start of the Discussion that more explicitly links the qualitative themes to the quantitative results and clarifies how the qualitative data helped to deepen interpretation of key statistical findings. We also draw on established mixed-methods guidance (e.g., Creswell & Creswell, 2017) to clarify the role of integration in explanatory sequential designs (see Page 22).<br><br>We believe this revision strengthens the transparency and coherence of the mixed-methods approach while remaining consistent with the study design.
	6	Results	Placement of Figure 1 disrupts manuscript flow. Figure 1, which maps survey findings to interview themes across the three research questions, is positioned within the Qualitative Findings section, following the first two thematic subheadings. As this figure is	Following the journal's formatting requirements, figures must now be submitted as separate files rather than embedded within the manuscript text. Accordingly, Figure 1 has been removed from the body of the manuscript and uploaded separately. In addition, the

			intended to provide an integrative overview of the entire thematic structure, its current placement mid-section and after partial presentation of themes means readers encounter it before having access to the full thematic content it summarises. This disrupts comprehension and reduces the figure's orienting function.<br>Figure 1 should be relocated to immediately precede the qualitative thematic findings section, so that it serves as a structural map before the themes are described in detail. Alternatively, if the journal format requires figures at the end, the in-text reference should clearly direct readers to consult the figure before reading the thematic descriptions.	journal requirements for figures specify a minimum resolution of 300 dpi and a size of 90 mm × 90 mm in width; accordingly, this figure has now been reformatted to meet these specifications.
Reviewer 2	1	Abstract	The abstract is clearly written and provides a good overview of the project and its results. One suggestion, though, would be to edit the Methods section of the abstract such that the ‘stress elements’ and the ‘key outcomes’ are phrased in the same manner as in the main document. That is, instead of ‘stressors’ and ‘appraisals’, ’work stressors’ and ‘stress appraisals’ should be used. Similarly, instead of ‘mental health’, ‘depression’ should be used.	We are pleased that the abstract was found to be clearly written and to provide a good overview of the study and its findings.<br><br>Regarding consistency of terminology in the abstract, we have revised the abstract accordingly to ensure alignment with the terminology used in the main manuscript, replacing “stressors” with “work stressors,” “appraisals” with “stress appraisals,” and “mental health” with “depression” where appropriate. We have replaced “mental health” with “depression” where referring specifically to the quantitative outcome measure to accurately reflect the

				instrument used. However, we have retained the broader term "mental health" where referring more generally to psychological outcomes discussed across the study (including qualitative findings), such as anxiety and related mental health experiences, as we believe this more accurately captures the wider range of constructs represented. In making these changes, we had to remove one word elsewhere in the abstract to remain within the journal's word limit (see Page 1).
	2	Introduction	The Introduction does a splendid job of clearly and succinctly explaining why the authors set out to perform a stress audit within ambulance staff.	We are delighted to receive this feedback, thank you for your positive comments and assessment of our manuscript. All your feedback has helped us improve the manuscript further.
	2a	Introduction	On line 28, there appears to be a typographical error in how the citation is presented to support the noted point.	This typographical error has now been corrected in the revised manuscript, and the citation formatting has been amended accordingly (see Page 3).
	2b	Introduction	In the final paragraph, the authors note that the stress audit was informed by two frameworks; although the first one of these (transactional stress theory) is described in an earlier paragraph, the same is not true of the second one (transdisciplinary model of stress). As a result, the authors should devote a few lines to informing the reader about the transdisciplinary model of stress and its relationship to the study.	We have expanded the manuscript to include a brief description of the transdisciplinary model of stress and its relevance to the present study. Specifically, we now clarify that this model conceptualises stress as arising not only from individual appraisal processes (as per the transactional stress theory), but also from broader contextual factors including organisational structures, workplace culture, and systemic influences (see Page 3).

	3a	Materials and Methods	On lines 10-12, the authors note that their survey was completed by 420 staff; a nice addition would be to inform the reader what proportion of the overall staff at this particular service is represented by those who participated.	We have now amended the manuscript to include the proportion of the eligible workforce represented by the sample. Specifically, we clarify that the survey was completed by 420 staff, representing approximately 7% of the eligible workforce at the service (see Page 4).
	3b	Materials and Methods	In Table 1, the authors note that they had 420 participants, yet when the number of participants in the 'sex' category are summed the result is 412.	We thank the reviewer for carefully checking the data in Table 1 and identifying this discrepancy. This was due to a typographical error in the reported value for Male. The correct total is 188 (not 180 as previously stated), and the table has now been corrected accordingly to ensure consistency with the overall sample size (see Page 4).
	3c	Materials and Methods	Carrying on with Table 1, the authors should consider changing 'sex' to 'gender', which is how they refer to this parameter through-out the rest of the manuscript.	We have amended Table 1 accordingly, replacing "sex" with "gender" to ensure consistency with the terminology used throughout the manuscript (see Page 4).
	3d	Materials and Methods	As well, in Table 1, the authors include a row for each category labelled 'missing', but its meaning is unclear. That is, are those labelled as 'missing' individuals on staff who chose not to participate (assuming that the total staff numbered 420), or are they those participants who did not enter a particular piece of information?	To clarify, the "missing" category in Table 1 refers to participants who took part in the survey (n = 420) but did not provide a response for that specific demographic variable. It does not refer to non-participants or non-responders to the study overall. We have clarified this in the revised manuscript/table notes to improve clarity and transparency (see Pages 4-5).
	3e	Materials and Methods	As a final point about Table 1, the authors should define or explain what 'bank' means within this context, for the benefit of international readers. Similarly, 'GDPR' (line 160) should be defined.	We have now clarified the meaning of "bank" in Table 1 by adding a definition in the table footnote. Specifically, we note that "bank" refers to paramedics employed on a flexible, shift-by-shift basis

				within the ambulance service, rather than on a fixed contracted rota, and typically used to cover staffing shortfalls or additional service demand (see Page 5). Similarly, General Data Protection Regulation (GDPR) has now be defined (See Page 5).
	3f	Materials and Methods	To help the reader appreciate a context for the results from this sample of ambulance staff, the authors should consider providing examples of the values that have been observed in the 'general population'. For instance, the average PHQ-2 score reported by the authors for their sample was 2.39 and it would be informative to see how this compares to what has been found in the broader population.	We agree that contextualising scores can aid interpretation. However, the PHQ-2 is primarily a brief screening instrument rather than a norm-referenced measure, and published "general population" averages vary considerably depending on sampling frame, weighting procedures, and survey context. As such, direct comparison risks being misleading and may not provide a stable or meaningful benchmark for interpretation in this occupational sample. Given that the primary aim of the study is to examine associations between stressors, psychological processes, and outcomes within ambulance staff rather than to estimate population prevalence or benchmark scores against external norms, we have therefore retained focus on within-sample interpretation and leave interested readers to make their own comparisons to general population statistics using existing literature should they wish.
	3g	Materials and Methods	Since Cohen's d is typically used as an effect size measure when considering the difference between two means, the authors may want to either explain, or include a citation to expand	We agree that Cohen's d is most appropriately used to quantify differences between two group means and is not the most suitable effect size metric for our linear regression models. In response, we have

			upon the application of Cohen's d to a linear regression model.	removed all Cohen's d values from Table 4 (now renumbered Table 3, see Page 13) and revised the manuscript accordingly (see Page 10). We now report standardised beta coefficients instead, which provide an appropriate measure of effect size within regression analyses in line with the guidance from the literature (Cohen, 1988; Peterson & Brown). [see Page 7]
	4a	Results	In Table 3, the mean for the wellbeing scale is presented as '39.94', yet the authors defined the scale as having 5 items, each with a maximum value of 5 (meaning that the maximum score would be expected to be 25). Please clarify.	The WHO-5 Wellbeing Index was scored using the standard transformation method, whereby the raw summed score (range 0–25) was multiplied by 4 to yield a percentage score ranging from 0 to 100. This transformation is consistent with the official WHO-5 scoring guidance and allows for easier interpretation, with higher scores indicating better wellbeing. We have now clarified this scoring approach in the Methods section (see Page 6) and updated the table notes to ensure transparency. Please note that, in accordance with the journal's table size requirements, Table 3 has been removed from the main manuscript and renumbered as Supplementary Table 1 in the Supplementary Materials.
	4b	Results	Similarly, the mean values presented for the demographic parameters do not seem appropriate. For example, the mean value for age is '2.92' and for gender is '1.54'.	We agree that reporting mean values for variables such as age category and gender coding may be misleading and not meaningful in their current form. In response, we have revised Table 3 to improve clarity and interpretability by removing demographic and work-characteristic variables

				from the correlation matrix. The table now focuses on the means, standard deviations, and intercorrelations among the key outcome and stress-related variables only. We believe this makes the table more succinct and improves its conceptual focus without loss of relevant information.<br>Please note that, in accordance with the journal's table size requirements, Table 3 has been removed from the main manuscript and renumbered as Supplementary Table 1 in the Supplementary Materials.
	4c	Results	With regards to Table 4, could the authors provide a brief explanation for their decision to include both the R2 and Cohen's d as measures of effect size? Also, were steps taken to determine whether bias existed in their residuals?	As noted in response to the previous comment 3g, we have now removed Cohen's d from all tables and the manuscript to ensure the appropriate use of effect size metrics for regression analyses. Table 4 (now Table 3, see Page 13) has therefore been revised to report R^2 as the primary measure of explained variance at the model level, alongside standardised beta coefficients to represent the magnitude of individual predictors.<br><br>Steps were taken to assess potential bias in the residuals and to evaluate whether key regression assumptions were met. We inspected standardised residuals and examined plots of residuals versus fitted values to assess homoscedasticity and detect any systematic patterns. We also reviewed normal probability (P–P) plots of the residuals to evaluate deviations from normality, and checked for influential observations using standard diagnostic statistics. These

				diagnostics tests did not indicate substantial violations of model assumptions or evidence of systematic bias in the residuals.
	4d	Results	For Table 5, would the authors consider including an effect size measure to allow these data to be presented in a manner consisted with those found in other tables?	We have now included omega-squared (ω^2) effect size estimates for the ANOVA analyses reported in Table 5 (now renumbered as Table 4). This reporting along with the effect size reporting for independent t-tests has also been clarified in the Methods (see Page 7).
	4e	Results	In Table 6, there are 346 degrees of freedom stated, which would allow a reader to assume that the sample size was 348 (given that an independent t-test was used). However, the authors report in Table 1 that there were 160 female participants and 180 male participants, which sum to a sample size of 340. Please clarify.	This issue relates to a typographical error previously identified by the reviewer in Table 1, where the number of male participants was incorrectly reported as 180 instead of 188. This has now been corrected in the revised manuscript. The corrected totals are consistent with the reported degrees of freedom in Table 6 (now renumbered as Table 5).
	4f	Results	Whilst lines 51-52 state that 'stress appraisal predicted wellbeing only', this does not seem to align with Table 4, which shows that stress appraisal predicts depression, wellbeing, and intention to leave scores. Please clarify.	We agree that the original wording in lines 51–52 was imprecise and may have been misleading when read alongside Table 4 (now renumbered Table 3). What we intended to convey is that stress appraisals showed their strongest and most substantial association with wellbeing, whereas mental rest demonstrated strong and consistent associations across all outcome variables (depression, wellbeing, and intention to leave), with large effect sizes observed across these outcomes. We have revised the manuscript text to clarify this distinction and

				ensure consistency with Table 4 (now renumbered Table 3, see Page 13).
	4g	Results	Certain items in the 'qualitative findings' section could be explained for the benefit of readers from outside the UK. In particular, the meaning of a 'delivery cell', 'rota', and 'bank staff'.	We agree that clarification would be helpful for an international readership. We have now defined "bank staff" and "rota" at their first appearances in the quantitative results section (see Page 16) and "delivery cell" in the Qualitative Section (see Page 17) to ensure clarity for readers unfamiliar with UK NHS staffing terminology.
	4h	Results	A small point, but the font size and clarity in Figure 1 could be enhanced to improve readability.	The journal requirements for figures specify a minimum resolution of 300 dpi and a size of 90 mm × 90 mm in width; accordingly, this figure has now been reformatted to meet these specifications which also improves clarity and readability. The figure is no longer embedded within the manuscript and is submitted as a separate file according to the journal's requirements.
	4i	Results	On page 18, the quotation highlighted in lines 37-44 nicely illustrates the point about suppressing emotions, however, the authors could more clearly connect the quotation to the preceding paragraph, which addresses the suppressing of emotions relating to stressors such as end-of-shift demands. In other words, the reader is presented with a quotation that clearly touches on stress caused by emotional suppression, but the quotation does not explain what caused the anger and frustration that the participant had to lock away.	We have revised the relevant section to more clearly link the quotation to the specific stressors being described in the preceding paragraph by explicitly stating the underlying causes of the emotional response (i.e., primary care call out). This amendment clarifies the context for the participant's reported anger and frustration and improves the interpretive flow between the narrative text and the illustrative quotation (see Page 19).

	5a	Discussion	On lines 49-51, (page 21) the authors state 'making real changes to daily organisational factors that impact ambulance staff is recommended'. Given the phrasing of the sentence, the reader can presume the authors intended that the 'impact' was positive, however, an 'impact' is not always positive. As a result, the authors may wish to edit the passage for clarity.	We agree that the original wording was potentially ambiguous, and have amended the sentence to clarify that we are referring to organisational factors that *adversely affect* ambulance staff. This change improves precision and removes any unintended implication of a neutral or positive impact (see Page 23).
	5b	Discussion	The discussion around 'mental rest' was interesting, and I wonder whether the authors would consider expanding the section to comment on what is known (if anything) about the perception and importance of 'mental rest' amongst other first responders (police and those in the fire service).	We have expanded the discussion to situate the construct of "mental rest" within the broader first responder literature. While the term itself is not widely standardised, we now clarify that it aligns with established concepts such as psychological detachment from work, recovery opportunities during shifts, and sleep-related recovery, which have been identified across fire and police populations as important factors influencing stress and wellbeing (see Page 23).
	5c	Discussion	Arguably, the most important sociodemographic variable is sex/gender. Given that the authors observed differences related to sex/gender, the Discussion section would be enhanced if they were to touch on these findings a bit more. For example, why might male staff be more at risk?	We agree that further elaboration of the observed gender differences strengthens the interpretation of the findings. In response, we have expanded the Discussion to more fully consider potential explanations for the higher risk observed among male staff. This now includes reference to possible differences in help-seeking behaviours, coping styles (e.g., greater use of avoidant or suppression-based coping), and occupational role patterns within ambulance services that may shape exposure to

				stressors and reporting of psychological outcomes (see Page 24).

Thanks once again for the insightful comments from the reviewers, we believe our manuscript has been improved further following their feedback.

References cited in response document:

Allen MS, Iliescu D, Greiff S. Single item measures in psychological science. European journal of psychological assessment. 2022 Jan 27.

Anchors ZG, Moore LJ, Burnard SD, Bressington CA, Moreton AE, Arnold R. A mixed-methods stress audit with midwives in the United Kingdom. Women and Birth. 2024 Sep 1;37(5):101639.

Braun V, Clarke V. To saturate or not to saturate? Questioning data saturation as a useful concept for thematic analysis and sample-size rationales. Qualitative research in sport, exercise and health. 2021 Mar 4;13(2):201-16.

Cohen J. *Statistical power analysis for the behavioral sciences*. 2nd ed. Hillsdale (NJ): Lawrence Erlbaum Associates; 1988.

Creswell JW, Creswell JD. Research design: Qualitative, quantitative, and mixed methods approaches. Sage publications; 2017 Dec 12.

Fugard AJB, Potts HWW. Supporting thinking on sample sizes for thematic analyses: a quantitative tool. *International Journal of Social Research Methodology*. 2015 Nov 2;18(6):669–84.

Hennink MM, Kaiser BN, Marconi VC. Code saturation versus meaning saturation: how many interviews are enough?. Qualitative health research. 2017 Mar;27(4):591-608.

Meese KA, Boitet LM, Sweeney KL, Gorman CA, Nassetta LB, Patel N, Rogers DA. Don't go: examining the relationships between meaning, work environment and turnover intention across the entire healthcare team. Journal of multidisciplinary healthcare. 2024 Dec 31:353-66.

Peterson RA, Brown SP. On the use of beta coefficients in meta-analysis. *J Appl Psychol*. 2005;90(1):175-81.

Schönbrodt FD, Perugini M. At what sample size do correlations stabilize?. Journal of Research in Personality. 2013 Oct 1;47(5):609-12.

VERSION 2 - REVIEW

Reviewer **1**

Name **Baek, Eun-Mi**

Affiliation **The Catholic University of Korea, Department of Preventive Medicine**

Date **07-Jun-2026**

COI

The authors have responded thoroughly and appropriately to the reviewers' comments and have revised the manuscript accordingly. The current version is well organized, scientifically sound, and suitable for publication in the journal. I have no further comments or recommendations for revision.

Reviewer **2**

Name **Mielke, John**

Affiliation **University of Waterloo, School of Public Health Sciences**

Date **04-Jun-2026**

COI

Thank you for the thoughtful attention given to the points raised in my review of the earlier manuscript draft.
